# Impacts of large herbivores on savanna plant communities: Predictive models of herbivore selectivity and plant response

**DOI:** 10.1002/ecy.70445

**Published:** 2026-07-23

**Authors:** Joel O. Abraham, Maria Stahl, Samson Kurukura, Abdikadir Ali Hassan, Jacob R. Goheen, Todd M. Palmer, Tyler R. Kartzinel, Robert M. Pringle

**Affiliations:** ^1^ Department of Ecology and Evolutionary Biology Princeton University Princeton New Jersey USA; ^2^ Department of Wildland Resources Utah State University Logan Utah USA; ^3^ Mpala Research Center Nanyuki Kenya; ^4^ Department of Natural Resource Ecology and Management Iowa State University Ames Iowa USA; ^5^ Department of Biology University of Florida Gainesville Florida USA; ^6^ Department of Ecology, Evolution and Organismal Biology and Institute for Environment and Society Brown University Providence Rhode Island USA; ^7^ Collegium Helveticum The Institute for Advanced Study of ETH, UZH, and ZHdK Zurich Switzerland

**Keywords:** DNA metabarcoding, elephants (*Loxodonta africana*), experimental ecological network analysis, keystone species, long‐term herbivore‐exclusion experiments, Mpala Research Centre (Laikipia County, Kenya), semiarid African savanna ecosystems

## Abstract

Large herbivores are among the most ecologically influential and extinction‐prone animals. Megaherbivores, in particular, radically alter vegetation. Yet, few studies have tried to predict the impacts of herbivores—or their extinction—on plant species composition and community structure. First principles suggest that preferred food plants should be most strongly suppressed by herbivores and released by herbivore loss, but this intuition may be misleading if plant responses are strongly contingent on functional traits and/or plant–plant interactions. We sought to predict the responses of plant species to size‐selective herbivore‐exclusion treatments in an African savanna, using data on herbivore diets and plant functional traits. Our analysis had three stages. First, we identified plant traits that predicted selectivity (use relative to availability) by the dominant herbivore species excluded by each experimental treatment: megaherbivores (elephant, giraffe; ≥1000 kg), mesoherbivores (buffalo, zebra, impala; 40–600 kg), and dik‐dik (5 kg). Several plant traits predicted selectivity across multiple herbivore species, but each species' diet was best predicted by a distinctive suite of traits. Second, we tested whether herbivore selectivity alone predicted plant responses. Elephant selectivity uniquely predicted plant responses in exclosures relative to unfenced control plots (*R* ^2^ = 0.24–0.30); plant taxa strongly favored by elephants were ninefold more abundant inside exclosures. However, herbivore selectivity failed to predict differences in community structure between the different fenced exclusion treatments, suggesting that bottom‐up effects of competition may intensify relative to consumptive effects as smaller herbivores are removed. Third, we show that including plant traits as covariates alongside elephant selectivity modestly improved predictability (*R* ^2^ = 0.27–0.50). Despite various sources of uncertainty and imprecision inherent in our approach, we show that elephant foraging decisions are a major determinant of plant community dynamics. Our findings indicate that models based on readily attainable data can substantially predict plant community responses to the loss or reintroduction of megafauna. Future work can refine our approach by incorporating additional plant traits associated with tolerance and competition, along with mechanistic measurements of herbivore preferences and biomass consumption, to predict even more accurately how large‐herbivore population declines and extinctions will impact plant communities.

## INTRODUCTION

Ecologists aspire to a predictive understanding of species interactions (Han et al., 2023; Maris et al., 2018; Maron et al., 2014). This quest has become more urgent in the context of anthropogenic global change. In both basic and applied research, forecasting the impacts of biodiversity loss—especially the loss of strongly interacting or keystone species that regulate community structure and ecosystem functions (Brose et al., 2017; Power et al., 1996; Zhao et al., 2016)—is a paramount challenge.

Large mammals are both disproportionately strong interactors and disproportionately prone to extinction. The body mass of wild mammal species has declined by an order of magnitude over the last 120,000 years (Smith et al., 2018) due to hunting, climate changes, and, lately, displacement by ~1 billion tons of combined human and livestock biomass (Greenspoon et al., 2023). Today, most large mammalian herbivores—including all but one species with an average adult body mass >400 kg (as per Smith et al., 2003)—are threatened or near‐threatened and occupy fragments of their historical ranges (IUCN, 2024; Ripple et al., 2015). Africa alone retains herbivore communities with a size range comparable to that of the Pleistocene megafauna (Smith et al., 2018), making African savannas especially valuable for studying the functional roles of large herbivores (Bakker et al., 2016; Pringle et al., 2023; Staver et al., 2021). Decades of research using fenced exclosures and large‐scale comparative analyses have shown that large herbivores influence myriad ecological variables, from soil and vegetation structure to bird and bee diversity, and that these impacts depend on herbivore body size: megaherbivores such as elephants, rhinos, and hippos exert unique effects that smaller ungulates cannot replicate (Augustine & McNaughton, 1998; Guy et al., 2021; Hobbs, 1996; Lundgren et al., 2024; Milchunas & Lauenroth, 1993; Owen‐Smith, 1988; Pringle et al., 2023; Trepel et al., 2024).

Some strong generalizations have emerged from this work, but most of them relate to coarse‐grained ecosystem‐level or community properties (e.g., plant biomass) and make correspondingly coarse‐grained predictions (Pringle et al., 2023). For instance, the densities of grazers and browsers, respectively, predict the impacts of herbivory on the overall abundance of grasses and trees (Karp et al., 2024; Staver et al., 2021). By contrast, few studies have tried to forecast more granular responses, such as plant community structure and species composition. Long‐term experiments are needed to develop and test such predictions, but most such experiments exclude all large herbivores simultaneously, making it difficult to isolate effects of particular herbivore species or assess how body size modulates ecological impact.

A key unanswered question is to what extent plant species' responses mirror herbivore foraging decisions. The simplest conceptual model is that any differences in abundance between exclosure and control treatments are purely attributable to herbivore offtake. Plants that are eaten in greater amounts—especially palatable species that are selected strongly even when rare—should be more strongly suppressed by herbivores and released by their removal. However, this model assumes that all plant taxa are equally sensitive to consumption. In reality, plants vary widely in tolerance and regrowth capacity (Strauss & Agrawal, 1999). Also, herbivore species differ in the lethality of their consumption. Ungulates primarily consume foliage, fruits, and stems, which is varyingly costly or even beneficial but rarely fatal, whereas elephants often cause mortality by severely damaging main stems or uprooting plants entirely (Abraham et al., 2021; O'Connor et al., 2007; Pringle et al., 2014). Thus, a more nuanced framework is that plant responses reflect a balance between herbivore selectivity, foraging mode, and plant tolerance (Augustine & McNaughton, 1998; Coley et al., 1985; Endara & Coley, 2011). Plant traits that confer tolerance to defoliation and/or structural damage should counterbalance effects of palatability. Consequently, a mix of avoided (well defended and/or nutrient‐poor) and selected (but fast‐growing, coppicing, or otherwise tolerant) plant taxa should predominate in the presence of herbivores, whereas palatable and/or intolerant taxa should proliferate inside exclosures. A still more complex prospect is that plant responses are an integrative function of herbivore selection, plant tolerance, and plant–plant interactions: herbivores exert direct effects via consumption and indirect effects by mediating competition and facilitation (Coverdale et al., 2016; Louthan et al., 2014).

Herbivore choice is a common ingredient in all of these scenarios, and its predictive power should be informative as to what limits plant populations. A tight relationship between herbivore selectivity and plant response would suggest strong and direct top‐down control; a weaker relationship would suggest a greater role of bottom‐up and indirect effects (tolerance, competition). Few studies have ever quantified this relationship. The dietary choices of mammalian herbivores are difficult to measure, and it remains unclear whether data on plant traits (which are easier to collect) can substitute for information on foraging behavior to predict plant population and community responses to herbivory (cf. Borer et al., 2019; Chollet et al., 2013; Cingolani et al., 2005; Evju et al., 2009; Milchunas & Lauenroth, 1993; Noy‐Meir et al., 1989).

Here, we tested whether detailed local dietary information enables prediction of plant responses to a decade of size‐selective herbivore exclusion in a semiarid Kenyan savanna. We used DNA metabarcoding (Kartzinel et al., 2019) and an extensive survey of plant abundance to estimate feeding selectivity (Jacobs's *D* index) for the commonest large herbivores, modeling herbivore selectivity as a function of plant traits. We then tested whether selectivity predicted the direction and magnitude of plant response to the exclusion of nested subsets of herbivore species: those >1000 kg, those >10 kg, and those >2 kg (Alston et al., 2022; Goheen et al., 2013). Last, we asked whether herbivore selectivity and plant traits together predicted plant responses better than selectivity alone. For each objective, we used model selection to identify the best predictive models based on the available data (Tredennick et al., 2021). Because our primary aim was prediction rather than explanation, and because the plant traits associated with properties such as tolerance/resilience and competitive ability are not well understood for African savannas, we refrain from overinterpreting the basis of trait relationships. This work is a first step towards a new framework that will enable researchers to mechanistically parse the roles of consumption, plant tolerance, and plant–plant interactions to predict species‐level responses of plant communities to large‐mammal herbivory.

We hypothesized that herbivore selectivity is predicted by plant traits associated with nutritional quality (e.g., protein, digestibility) and antiherbivore resistance (e.g., spines, leaf toughness), and that the traits predictive of selectivity differ for each herbivore species given their distinctive diets (Kartzinel et al., 2015, 2019; Pansu et al., 2022). We also hypothesized that the selectivity of elephants (*Loxodonta africana*), impala (*Aepyceros melampus*), and dik‐dik (*Madoqua guentheri*) best predict the response of plant populations to the removal of herbivore assemblages, because these species have the highest biomass densities at our study site, and because elephants in particular have extraordinary impacts on plants (Abraham et al., 2021; Asner et al., 2009; Coverdale et al., 2024; Laws, 1970). Finally, we hypothesized that herbivore selectivity would predict the bulk of the variance in plant responses and that the remainder would stem from species‐ and site‐specific variation in tolerance and plant–plant interactions. Accordingly, we expected that plant traits linked to tolerance and competitive ability would predict some of the residual variance in response to herbivore exclusion; however, we were unable to specify a priori which traits these might be, as regrowth capacity and competitive hierarchies can involve many plant traits in complex, context‐dependent ways.

## MATERIALS AND METHODS

### Study site and experimental design

**FIGURE 1:**
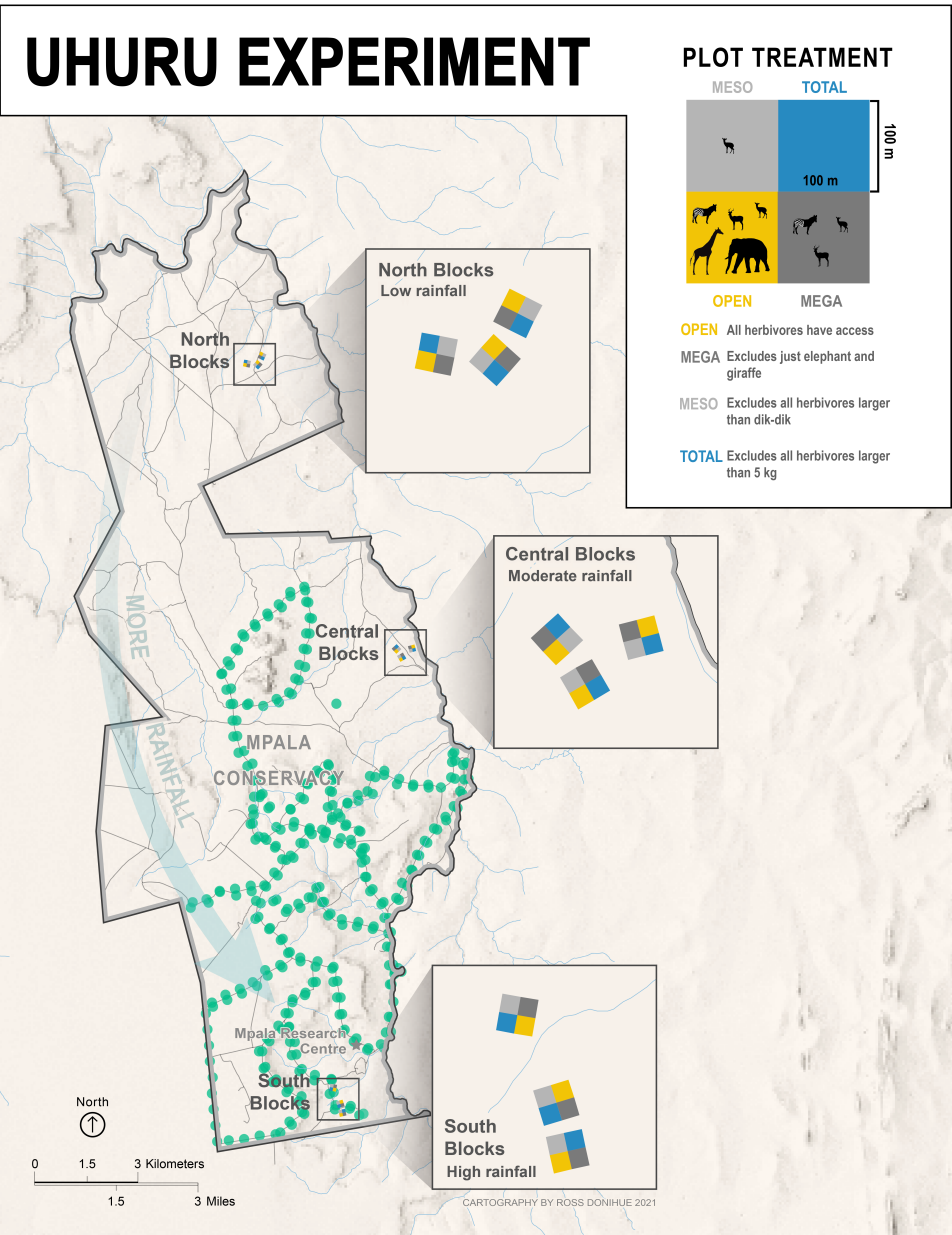
Study design, Laikipia, Kenya. The UHURU experiment at Mpala Research Centre (0°17′ N, 36°54′ N) comprises 36 1‐ha plots: three replicate blocks of four treatments (OPEN, MEGA, MESO, TOTAL) at each of three sites (south, central, north) across a rainfall gradient. Pairs of green circles show the locations (*N* = 326, on opposite sides of dirt roads) where we measured the relative availability of plants to estimate herbivore foraging selectivity. Animal silhouettes by Rob Pringle. Map and schematic design by Ross Donohue.

The Ungulate Herbivory Under Rainfall Uncertainty (UHURU) experiment (Figure 1) is located at Mpala Research Centre (0°17′ N, 36°52′ E), a 200‐km^2^ ranch in semiarid central Kenya. From 2009 to 2019, Mpala averaged 560 mm year^−1^ (±143 SD) of rainfall (60 ± 13 days with ≥1 mm). Most rain falls from April to November with a dry season from December to March (Alston et al., 2022; Goheen et al., 2013). UHURU spans a rainfall gradient, with ~15% higher total annual rainfall in the south (600 mm) than 20 km farther north (520 mm) from 2008 to 2019 (Alston et al., 2022). The overstory is dominated by spiny acacia species (*Vachellia etbaica*, *Senegalia brevispica*, *S. mellifera*), with a diverse and discontinuous understory of grasses, forbs, and subshrubs (Gill et al., 2019; Goheen et al., 2013).

Over 20 species of large herbivore occur at Mpala, ranging in size from 5 kg (dik‐dik, *M. guentheri*) up to 5000 kg (bull elephant, *L. africana*). Livestock have been kept in decreasing numbers over the last decade (Titcomb et al., 2022). Systematic data on herbivore species' densities do not exist for recent years, but distance‐sampling estimates from 2000 to 2002 (Augustine, 2010) are broadly congruent with data from dung counts in UHURU (Alston et al., 2022) and with data for assorted species estimated by distance sampling from 2007 to 2014 (Ford et al., 2015; Pringle et al., 2016), so we consider them a valid reference point. Elephants have the highest biomass (1.7 individuals and 2900 kg km^−2^), dik‐dik are most numerous (140 individuals and 700 kg km^−2^), and impala (*A. melampus*) are also abundant (20 animals and 800 kg km^−2^). Giraffe (*Giraffa camelopardalis*), buffalo (*Syncerus caffer*), and zebras (*Equus quagga*, *E. grevyi*) are also common but have biomass densities 1–2 orders of magnitude lower (Augustine, 2010) and dung densities 2–4‐fold lower (Alston et al., 2022) than the three dominant species.

UHURU comprises 36 1‐ha plots replicated in blocks (Goheen et al., 2013). Each block has four contiguous plots, with three exclosure treatments and an unfenced control. There are three blocks at each of three sites in the south, center, and north of Mpala (Figure 1). OPEN plots allow all animals. MEGA plots exclude only two megaherbivores, elephant and giraffe, using 2‐m high electric wires. MESO plots exclude megaherbivores and mesoherbivores (impala, zebra, buffalo, and other midsized ungulates) using wires down to nearly ground level. TOTAL plots exclude all ungulates using electric wires and chain link, but allow rodents and hares (≤2 kg). UHURU was established in 2008, and raw data through 2019 are publicly available (Alston et al., 2022).

### Measuring plant response to herbivore exclusion

The central 60 × 60 m of each UHURU plot is subdivided into a grid of 10 × 10 m cells demarcated by 49 stakes. We assessed vegetation composition at each stake using a modified canopy‐intercept approach. Minor variations on the classic canopy‐intercept method (Frank & McNaughton, 1990) have been used for decades to estimate herbaceous cover and biomass, where researchers pass wire pins through the understory and count the number of contacts (“pin hits”) per plant species. We adapted this method by using one giant pin to quantify the relative abundance and species composition of all understory and overstory plants in the same units (number of pin hits), as is necessary to characterize the selectivity and community‐wide impacts of mixed‐feeding large herbivores such as impala and elephant (Alston et al., 2022; Coverdale et al., 2021; Kartzinel & Pringle, 2020). We placed a telescoping measuring pole on the ground and extended it upwards, recording the number and height of all pin hits from ground level to the top of the tallest tree. In a full survey, we repeated this process at all 49 grid stakes in all 36 UHURU plots. Here, we used data from three full surveys (March 2017, December 2018, October 2021), which encompassed marked seasonal and interannual variation in rainfall (16–116 mm in the 90 days up to the end of each survey; 192–574 mm year^−1^). A few stakes were omitted in each survey; we replaced these with data from partial surveys conducted in October 2016 and April 2018. All 1764 stakes are thus represented exactly three times in our dataset (total effort = 5292 pins).

For each plant taxon, we summed the number of records across the three surveys of each plot (aggregating across repeated measures) and then across the three blocks of each treatment at each site (aggregating across spatial replicates), yielding one relative‐abundance estimate per treatment per site. This process lumps nine true replicates of each treatment into three site‐level units of analysis to reduce noise and idiosyncrasy, which can bias log‐response ratios (see *Statistical analysis*).

Pin‐hit data are tightly and linearly correlated with dry biomass, but slopes often vary by growth form (Frank & McNaughton, 1990). At Mpala, for example, biomass (in grams per square meter) increased with pin hits more steeply for bunchgrasses and forbs (42×–52×) than for thin‐leaved grasses (26×; Augustine, 2003). A similar calibration to convert our data into absolute biomass would be very difficult and is nonessential provided that our method preserves species' relative abundances. We conducted several analyses to validate this assumption (Appendix S1: Figure S1; Appendix S2). First, we reanalyzed data digitized from Frank and McNaughton (1990) and found that the biomass ~ pin hits correlation was strong even when we ignored plant growth form (*r* = 0.82) and stronger still when we converted both variables to ranks (*r* = 0.91, Spearman's ρ = 0.91). Next, we compared the data with contemporaneous (2017–2018) data from tree censuses and understory surveys (percent cover, pin frame) in the same experimental plots (Alston et al., 2022). Although there was variation between methods, particularly for low‐abundance taxa, data were generally highly correlated across methods (*r* = 0.68–0.98; ρ = 0.69–0.74), indicating that our vegetation sampling method reproduces patterns of relative abundance across plant functional types.

### Quantifying relative forage availability

Quantifying herbivore selectivity requires estimating the relative abundance of plants in both diets and the environment. To measure plant relative availability independently of the experimental data, we used our pin‐intercept method to survey vegetation throughout Mpala from July to September 2017 on the same 83 km of dirt road where we collected herbivore fecal samples for diet analysis (Figure 1). We stopped at 0.5‐km intervals, chose random distances (0–50 m) from both sides of the road, walked off those distances, and measured vegetation as described above (*N* = 326 locations, 3387 pin hits, 109 plant species). We calculated overall relative availability by dividing the number of records of each species by the total number of records, and we also derived grass‐ and nongrass‐specific relative availabilities.

To probe the adequacy of these data for estimating selectivity, we compared them to the larger sample of giant‐pin hits in unfenced OPEN plots (1323 total pins; Appendix S2). Despite the different spatial extent and distribution of sampling in these two surveys (Figure 1), plant relative‐abundance estimates were strongly correlated (*r* = 0.64–0.71; Appendix S1: Figure S2), albeit with some scatter, particularly for relatively rare taxa. These relationships were similar for grasses, nongrasses, and all taxa combined, with no evidence of bias related to species' abundances or growth forms. Moreover, sample‐based rarefaction and extrapolation showed that observed species richness was well past the inflection point in the accumulation curve (Appendix S1: Figure S3). While additional sampling would thus have detected additional plant species, these undetected plants represent a minority of herbivore diets: the median dietary relative abundance of individual undetected plants was 0.2% (range = 0.1%–6.2%), and the median cumulative abundance of all undetected plants within a herbivore species' average diet profile was 12.8% (range = 8.2%–25.5%; Appendix S1: Figure S4).

### Characterizing plant traits

From June to August of 2016 and 2018, we collected data on physical and chemical traits for over 300 of the ~500 plant species at Mpala, including all species that are common in the landscape and in herbivore diets. We measured 41 traits but used only 15 weakly correlated traits in our analyses, as described below (Appendix S1: Figure S5). We dropped the 26 other traits because of gaps in species coverage, redundancy or high collinearity with other traits, presence of extreme or otherwise dubious values, or lack of a hypothesis for the ecological relevance of the variable. All trait data are available in the data repository associated with this paper (Abraham et al., 2026).

We coded spinescence categorically (i.e., whether the plant species produces spines, prickles, or thorns). To measure leaf toughness, we collected three fresh but mature leaves, cut strips 2‐cm long (parallel to midrib) and 1–3 mm wide (perpendicular), and measured force to tear with a 50 N compact gauge (Mecmesin, West Sussex, UK); we then standardized by dividing by strip width. For plant height, we used the 97.5^th^ percentile value recorded in the field surveys described in the preceding subsections. This variable thus integrates across demographic stages and reflects species' maximum stature under field conditions (it is not a surrogate for herbivore foraging height; it simply indexes how tall each plant species can grow). Lastly, we pooled young leaves from multiple adult individuals per species and submitted dried samples to a commercial laboratory (Crop Nutritional Services, Nairobi, Kenya; Complete Feed Analysis). We used data on digestibility (percent neutral cellulase plus gammanase digestibility from near‐infrared reflectance spectroscopy); percent fiber (acid and alkaline digestion, ISO 6865) and protein (sulfuric acid digestion, ISO 8968‐1); and concentrations of nine minerals (atomic emissions spectrometry, AOAC 985.01): calcium (Ca, %), copper (Cu, ppm), iron (Fe, ppm), potassium (K, %), magnesium (Mg, %), molybdenum (Mo, ppm), sodium (Na, ppm), phosphorus (P, %), and zinc (Zn, ppm).

### Inferring herbivore diet composition

We used our own previously published diet data from fecal DNA metabarcoding at Mpala over 4 years (2013–2016), spanning wet and dry periods (Kartzinel et al., 2019). These data include an average of ~100 samples for each of the six most common herbivores (80 elephant, 58 giraffe, 92 buffalo, 91 Grevy's zebra, 106 plains zebra, 129 impala, and 120 dik‐dik; 9–48 samples species^−1^ year^−1^). All data and field, laboratory, and informatic protocols are in Kartzinel et al. (2019) and associated repositories. We amplified the P6 loop of the chloroplast *trn*L (UAA) intron (Taberlet et al., 2007) and sequenced libraries as single‐end 170‐bp reads on an Illumina HiSeq 2500 at Princeton University. We identified sequences (molecular operational taxonomic units, mOTUs) using a nearly comprehensive local reference database comprising thousands of specimens, 460 species and 312 unique *trn*L‐P6 sequences (Freeman et al., 2022; Gill et al., 2019). Most mOTUs uniquely matched just one locally occurring plant species or genus; 24 mOTUs matched at least two and as many as 12 species from different genera in the same family. To harmonize taxonomic resolution, we coarsened identifications in the plant survey data to match those in the diet data. For example, the forbs *Indigofera schimperi* and *I. hochstetteri* were lumped into a subgeneric composite taxon, as were the grasses *Digitaria macroblephara* and *D. velutina*. For plant survey data, we summed records for all species subsumed in a given mOTU (stratified by site and treatment in UHURU). For most trait data, we averaged across all species for which we had measurements; for height, we used the 97.5^th^ percentile across all observations of species within an mOTU. Analyses of mineral nutrient traits showed that the degree of trait variation between species in the same mOTU was much smaller than that between species in different mOTUs (Appendix S1: Figures S6 and S7), indicating that multispecies mOTUs are ecologically coherent units consisting of species with similar traits.

Following Kartzinel et al. (2019), we used relative read abundance (RRA)—the proportional representation of each mOTU after rarefying to a common read depth—to characterize diet composition. We averaged the RRA of each mOTU across all samples from each herbivore species to obtain time‐ and space‐integrated diet profiles. RRA is an imperfect proxy for intake, because primer mismatches, amplification biases, and differential digestibility introduce noise (Deagle et al., 2019). That said, multiple lines of evidence indicate that mean RRA across our large sample sizes is broadly representative of species‐level patterns in consumption. These include feeding trials (Willerslev et al., 2014), computer simulations (Littleford‐Colquhoun et al., 2022), and stable‐isotope analyses at Mpala (Kartzinel et al., 2015) and elsewhere (Craine et al., 2015). Our confidence is also based in part on inferences from our extensive prior work using this method, where we have repeatedly found that trends in RRA correspond with foraging observations, shifts in plant phenology, patterns of herbivore space use in relation to plant availability, and theoretical predictions (Atkins et al., 2019; Branco et al., 2019; Daskin et al., 2023; Guyton et al., 2020; Kartzinel et al., 2019; Kartzinel & Pringle, 2020; Pansu et al., 2019, 2022; Potter et al., 2022; Walker, Hutchinson, Becker, et al., 2023; Walker, Hutchinson, Potter, et al., 2023). Along similar lines, we found here that plant relative availability was correlated with mean RRA, explaining up to 50% of the variance across herbivore species (Appendix S1: Figure S8). This correlation is expected, and thus an encouraging sign of fidelity in our plant and RRA data, because intake inherently depends on availability. However, it has potential to confound the interpretation of relationships between herbivore diet composition and plant response to herbivory.

In theory, plant response should depend more on herbivore preference (what they select even when rare) than consumption (highly abundant plants may be frequently eaten but weakly responsive if offtake is a small fraction of standing biomass). Accordingly, we used selectivity for each plant taxon as our main predictor, calculated as Jacobs's (1974) *D* index:

$$D_{i}=\frac{d_{i}-a_{i}}{d_{i}+a_{i}-2d_{i}a_{i}},$$

where *D*_*i*_ is selectivity for plant taxon *i*, *d*_*i*_ is the proportion of plant *i* in the diet, and *a*_*i*_ is its availability in the environment. *D* ranges from −1 to 1; positive values indicate selection (more common in diet than environment) and negative values indicate avoidance (relative to availability).

We calculated *D* values for the six most abundant herbivore species, which collectively account for ~90% of herbivore biomass (Augustine, 2010) and have complementary feeding habits *vis a vis* the UHURU treatments (Appendix S1: Table S1). These included the three dominant species—elephant (140 mOTUs), impala (151 mOTUs), and dik‐dik (142 mOTUs), which are targeted by the MEGA, MESO, and TOTAL treatments, respectively—along with three subdominant species: giraffe, which browse trees and are the only species besides elephant excluded by MEGA (114 mOTUs); buffalo, a mixed feeder on grasses and forbs, excluded by MESO (146 mOTUs); and the two zebra species (105 mOTUs), whose diets we averaged because they are numerically and functionally similar (Abraham, Lin, et al., 2024; Kartzinel et al., 2015). For strict grazers (zebra) and browsers (giraffe, dik‐dik), we also estimated grass‐ and nongrass‐specific *D* values using grass‐ and nongrass‐specific relative availabilities (Appendix S1: Table S1).

For additional validation of the availability data for short‐statured plants, and because dik‐dik cannot forage higher than 1‐m, we calculated an alternative *D*_dik_ metric based only on availability from pin hits ≤1‐m high. This metric was essentially identical to that based on overall availability (*r* > 0.99; Appendix S1: Figure S9, Table S1) and did not affect any results (Appendix S1: Tables S2 and S3), so we present only results using overall selectivity. We omitted plant mOTUs that were not detected in any plant survey, which accounted for a small fraction of dietary RRA (≤6.2%: Appendix S1: Figure S4), along with those not detected in the diet of any herbivore (Kartzinel et al., 2019). This yielded 65 estimates of *D* per herbivore species (15 grass and 50 nongrass mOTUs), which ranged from −1.00 to 0.95 (Appendix S1: Table S1).

### Statistical analysis

We calculated the log‐response ratio of each plant taxon for each pair of herbivore‐exclusion treatments, ln(*N*_*i,E*_/*N*_*i,C*_), where *N*_*i*_ is the number of pin hits for plant *i*, *E* is the more exclusive of the two treatments, and *C* is the “baseline” treatment. We studied five contrasts, three of which used OPEN as the baseline: megaherbivore exclusion (MEGA:OPEN); mega‐ and mesoherbivore exclusion (MESO:OPEN); and exclusion of all large herbivores (TOTAL:OPEN). We also analyzed the exclusion of mesoherbivores only (MESO:MEGA) and of dik‐dik only (TOTAL:MESO). The log response ratio measures effect size but is sensitive to small numbers (Hedges et al., 1999); thus, in addition to pooling data across replicate plots in each site, we calculated responses only for taxa that occurred in both treatments and with ≥5 hits in one of them.

We built candidate sets of generalized linear models for three categories of dependent variable. First, we tested which plant traits predicted selectivity: for each herbivore species, we modeled *D* as a function of the 15 chosen plant traits (Appendix S1: Table S2). Second, we tested the extent to which herbivore selectivity alone predicted plant response, modeling effect sizes as functions of herbivore *D* estimates (Appendix S1: Table S3). Third, we tested whether including plant traits improved these predictions (Appendix S1: Table S4): for the same treatment contrasts, we tested plant traits together with elephant selectivity (*D*_ele_), which was the best predictor in the preceding analysis (see *Results*). Trait data were standardized and centered prior to analysis. For completeness, we repeated the first two stages of these analyses using both frequency of occurrence (FOO; Appendix S1: Tables S5 and S6) and RRA (Appendix S1: Tables S7 and S8) in lieu of selectivity, but we present only the selectivity‐based models here due to our concern about the potentially confounding effects of availability.

For each stage of our analyses, we built a global model that included all additive combinations of all predictors. Models of plant response included a random intercept for site to account for spatial heterogeneity and instances where plant taxa occurred in the same treatment contrast at multiple sites. We fit models in R (v4.3.2; R Core Team, 2024) using lme4 (v.1.1.35.1; Bates et al., 2015). Variance inflation factors were <7 for all predictors. As an added precaution against overfitting, we excluded candidate models containing any pair of predictors with |*r*| > 0.7, while retaining all predictors in each candidate model set (Appendix S1: Figure S5). We then fit all remaining subsets of the global models and ranked them based on the Akaike information criterion (AIC_c_), using *dredge* in MuMIn (v1.47.5; Bartoń, 2023). We focus on models with ΔAIC_c_ < 2 (2AIC_c_), a widely used quality threshold (Anderson & Burnham, 2002; Grueber et al., 2011). We inspected residuals for signs of misspecification and identified the most frequent and consistent predictors of each response. To aid visualization and summarization, we conditionally averaged coefficients and determined associated SEs across the top 2AIC_c_ models. We calculated relative variable importance as the sum of Akaike weights of all models with that predictor. The top models are in Appendix S1; full model tables are in the Dryad Digital Repository along with all data and R scripts (Abraham et al., 2026).

## RESULTS

### Patterns and predictors of herbivore selectivity

Elephant, giraffe, buffalo, impala, and dik‐dik exhibited correlated patterns of selectivity (Pearson's *r* = 0.24–0.78, Spearman's 𝜌 = 0.37–0.74); zebra were outliers owing to their strict specialization on grasses (Appendix S1: Figure S5). Across herbivore species, *D* was reasonably well predicted by combinations of up to seven plant traits (median *R* ^2^ [*R* ^2^_med_] across the top 2AIC_c_ = 0.12–0.67). Model fits were generally better for strict grazers and browsers (zebra, dik‐dik, and giraffe; *R* ^2^_med_ = 0.38–0.62 overall; *R* ^2^_med_ = 0.12–0.67 for grass‐/nongrass‐specific model sets) than for mixed feeders (impala, buffalo, and elephant; *R* ^2^_med_ = 0.14, 0.18, and 0.38, respectively).

**FIGURE 2:**
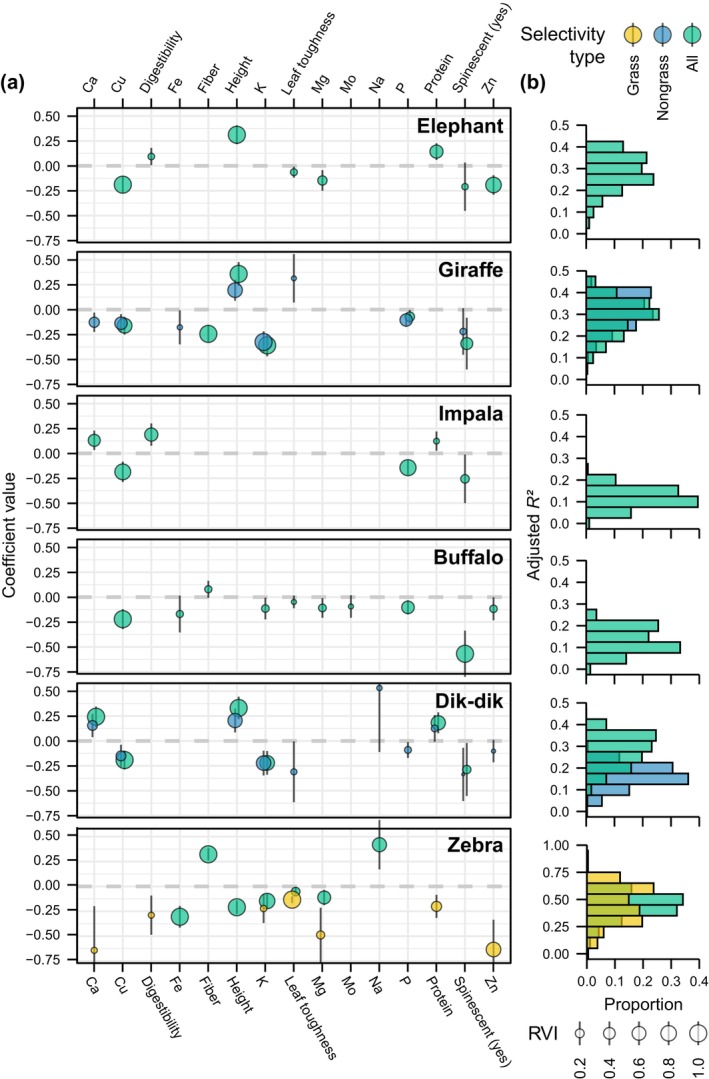
Predictors of herbivore selectivity (*D*) for different plant taxa. (a) Conditionally averaged coefficients for models in the top 2AIC_c_ (see Appendix S1: Table S2). Points are scaled by relative variable importance (RVI; summed Akaike weights, or relative likelihoods, across all models including a given predictor). Colors show whether selectivity was measured based on the availability of all plant taxa (green; *N* = 65 taxa and *D* estimates), all taxa except grasses (blue; *N* = 50), or grasses only (gold; *N* = 15). Whiskers show ±1 coefficient SE. (b) Proportional distribution of *R* ^2^ values across all models tested (note the different *y*‐axis scale for zebra). AIC_c_, corrected Akaike information criterion.

A few traits had consistently positive or negative effects on selectivity across herbivore species (Figure 2 and Appendix S1: Table S2). Copper concentration was the single most general predictor of *D*, with negative coefficients indicating that plant taxa with lower Cu concentrations were more strongly selected relative to Cu‐rich taxa by browsers and mixed feeders (Figure 2). A cluster of other elemental nutrients (P, K, Zn, Mg, Fe) occurred less consistently in the top 2AIC_c_ but always had negative coefficients, likewise indicating inverse relationships between foliar concentrations and herbivore selectivity. Spinescence was another consistently negative predictor of *D* (most importantly for buffalo), whereas protein was an important positive predictor for elephant and dik‐dik (Figure 2). Other patterns reflected generic differences between grasses and nongrasses in traits such as height and fiber content: elephant, giraffe, and dik‐dik selected for taller taxa (trees and shrubs), but zebra did the opposite; zebra and buffalo selected for more fibrous taxa (grasses), but giraffe did the opposite. Use of grass‐ and nongrass‐specific relative availabilities for grazers and browsers muted these trends: fiber became irrelevant, and height and spinescence declined in relative importance (Figure 2). For zebra, grass‐specific selectivity boiled down to leaf toughness and zinc (*R* ^2^ = 0.64), with negative coefficients reflecting selection for relatively tender, Zn‐poor taxa such as *Brachiaria*, *Setaria*, and *Harpachne* spp. and relative avoidance of tougher, Zn‐rich taxa such as *Pennisetum* spp. (Appendix S1: Tables S1 and S2).

Despite these consistencies in the effects of several plant traits across herbivore species, we also found evidence of interspecific differentiation. No two species had the same predictors in the same order of importance (Figure 2). For example, although elephant, giraffe, and dik‐dik all selected first and foremost for relatively tall‐statured plant taxa (reflecting the prevalence of trees and shrubs in their diets), the next most important variables differed for each species: Cu (negative) for elephant, K (negative) for giraffe, and Ca (positive) for dik‐dik.

### Does herbivore selectivity predict plant response?

**FIGURE 3:**
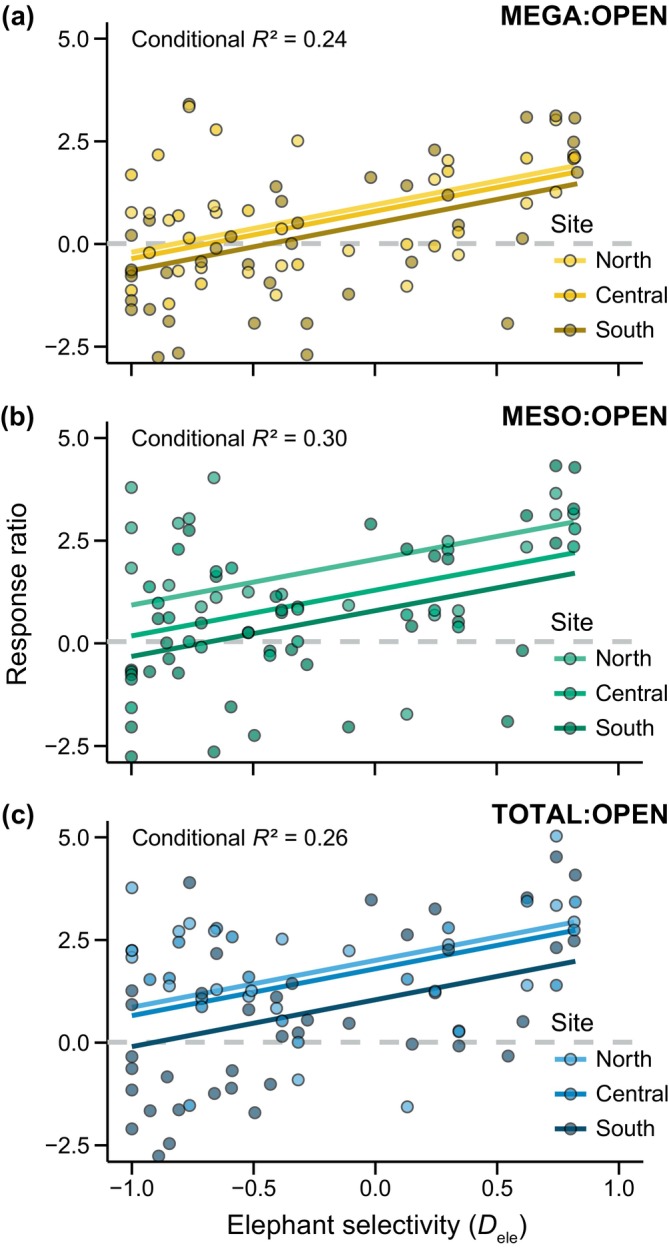
Elephant selectivity predicts plant responses to herbivore exclusion. (a–c) Log response ratios for each treatment, relative to unfenced control plots, as a function of elephant selectivity, *D*_ele_ (see Appendix S1: Table S3). Each point corresponds to a plant taxon (species or suite of closely related species; see *Materials and Methods*) at one of the three sites (south, central, north). Fitted lines show models with *D*_ele_ as the sole fixed effect and random intercepts for each site.

Elephant selectivity (*D*_ele_) strongly predicted plant responses in all exclosure treatments relative to unfenced OPEN plots (𝛽 = 1.00–1.27, conditional *R* ^2^ = 0.24–0.30; Appendix S1: Table S3). All but a few of the 28 plant taxa selected by elephants (*D*_ele_ >0) were more abundant in plots without elephants; the most strongly selected taxa (*D*_ele_ >0.5) were ninefold more abundant on average in exclosures, whereas those that were strongly avoided by elephants (*D*_ele_ < −0.5) were on average equally abundant in exclosures and OPEN plots (Figure 3). The top 2AIC_c_ for these contrasts included models featuring *D*_ele_ along with negative effects of *D*_zeb_ and *D*_buf_, indicating that the grasses selected by zebra and buffalo tended to decline within exclosures at the expense of woody plants that they avoid. However, these effects were weaker than those of *D*_ele_ and only marginally enhanced model fits (Appendix S1: Table S3). Notwithstanding the confounding effects of availability, FOO‐ and RRA‐based models produced broadly similar results (Appendix S1: Tables S5 and S7).

In contrast, plant responses to the stepwise removal of mesoherbivores relative to plots without megaherbivores (MESO:MEGA) and of dik‐dik relative to plots without mesoherbivores (TOTAL:MESO) were not well predicted by any combination of *D* (Appendix S1: Table S3). In both cases, the null model [intercept + (1|site)] was the best in the set (∆AIC_c_ ≥ 1.78 and 2.85, respectively).

### Does incorporating trait data improve predictions of plant response?

**FIGURE 4:**
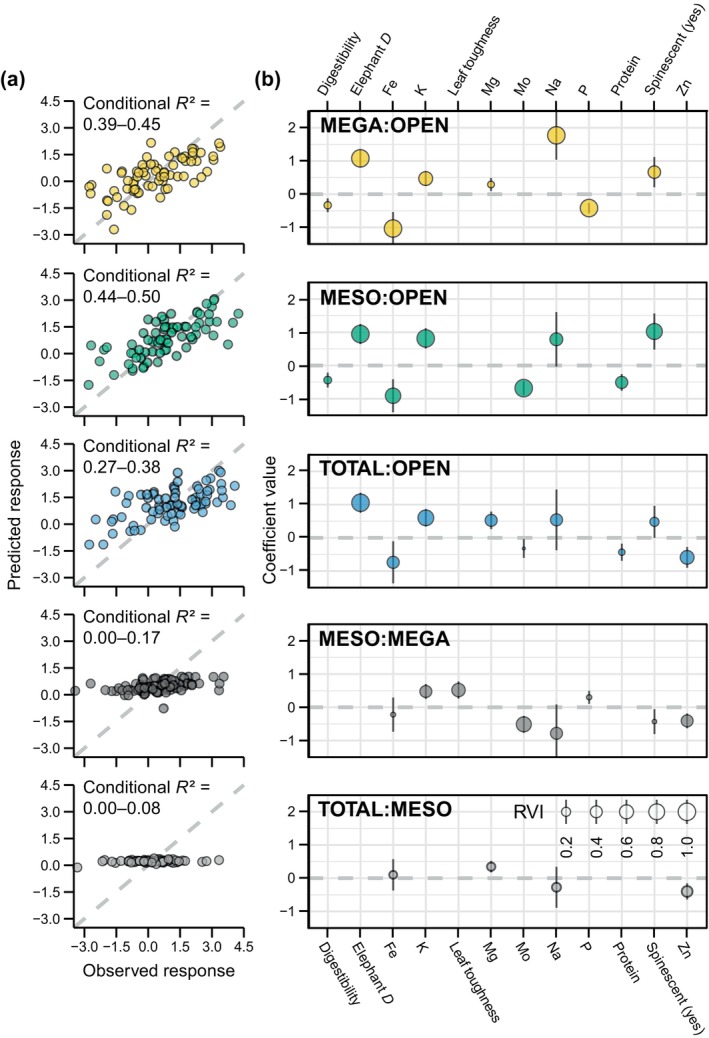
Plant traits enhance predictability of plant response to herbivore exclusion. (a) Observed versus predicted plant‐response values from conditionally averaged model coefficients, as well as the range of conditional *R* ^2^ values for the top 2AIC_c_ models (for comparison against *D*_ele_‐only conditional *R* ^2^ values in Figure 3). Colors denote treatment contrasts. Dashed gray lines show 1:1 correspondence, where predicted and observed responses match. (b) Averaged model coefficient values for each predictor, as used in (a). Point sizes are scaled by relative variable importance, as in Figure 2a. Whiskers show ±1 coefficient SE. AIC_c_, corrected Akaike's information criterion.

Including plant traits as predictors together with *D*_ele_ modestly improved predictions of plant response (Figure 4 and Appendix S1: Table S4). *D*_ele_ remained an essential predictor and was retained in all models in the top 2AIC_c_ for all contrasts between exclosure treatments and OPEN plots. Beyond that, a subset of traits, mostly mineral nutrients, enhanced predictions to varying degrees. Among the most consistent of these were K and Fe: the former was positively related to plant response in exclosures (plant taxa with higher foliar K tended to increase more in exclosure treatments), whereas the latter had consistently negative effects (plants with higher Fe tended to decline more with herbivore exclusion). Other traits—Mo (negative), Na (positive), P (negative), and spinescence (positive)—were key predictors for some treatment contrasts but not others.

*D*_ele_ was the only predictor that featured in all 31 top models for TOTAL:OPEN (*R* ^2^ = 0.27–0.38). The best model included positive effects of *D*_ele_ and K and a negative effect of Fe; Na (positive), Mg (positive), and Zn (negative) also featured prominently in the top 2AIC_c_. For MESO:OPEN, all 11 top models included positive effects of *D*_ele_ and K and negative effects of Mo, and all but two also included positive effects of spinescence; Na (positive) and Fe (negative) were also common (*R* ^2^ = 0.44–0.50). For MEGA:OPEN, all six top models included positive effects of *D*_ele_ and Na along with negative effects of Fe and P; half of these models also included positive effects of K and/or spinescence (*R* ^2^ = 0.39–0.45). Other predictors included in the top 2AIC_c_ for these contrasts were relatively unimportant (Figure 4b; Appendix S1: Table S4). The fits of these models are not directly comparable to those with *D*_ele_ alone, as missing trait data forced us to exclude some plant taxa. However, thinning the latter data to match indicated that including traits increased *R* ^2^ by up to 0.22 for MEGA:OPEN (*R* ^2^ = 0.39–0.45 vs. 0.23), 0.17 for MESO:OPEN (*R* ^2^ = 0.44–0.50 vs. 0.33), and 0.11 for TOTAL:OPEN (*R* ^2^ = 0.27–0.38 vs. 0.27). Similarly, relative to the best fitting traits‐only model in each model set, adding *D*_ele_ increased *R* ^2^ by up to 0.14 for MEGA:OPEN (*R* ^2^ = 0.39–0.45 vs. 0.31), 0.11 for MESO:OPEN (*R* ^2^ = 0.44–0.50 vs. 0.39), and 0.14 for TOTAL:OPEN (*R* ^2^ = 0.27–0.38 vs. 0.24), indicating that elephant selectivity confers predictive power not captured by plant traits.

Including traits did not appreciably enhance predictability for the stepwise contrasts involving “modified baselines.” For TOTAL:MESO (*N* = 78), the null model remained the best in the set (∆AIC_c_ ≥ 1.20). For MESO:MEGA (*N* = 74), 21 models were within 2AIC_c_, with the null ranked 12th among these. Leaf toughness (positive) and Mo (negative) were included in more than half of the top models, and the 2nd‐ranked model (positive effects of leaf toughness and K, negative effects of Mo and Na) accounted for 15% of the variance with seven parameters, but none of these models approached the predictability of contrasts between exclosure treatments and OPEN plots.

## DISCUSSION

Predicting the effects of large herbivores on plants is a goal common to community ecology, paleoecology, rangeland management, biodiversity conservation, and restoration (Bakker et al., 2016; Milchunas & Lauenroth, 1993; Svenning et al., 2016). It is known that herbivore selectivity differs among plant taxa (some plants are more readily consumed than others) and that plants differ in their numerical response to herbivory (herbivores alter plant community structure). As to what extent the former drives the latter, however, our understanding has not progressed much beyond conceptual models published over 25 years ago (Augustine & McNaughton, 1998; Coley et al., 1985; Milchunas et al., 1988; Noy‐Meir et al., 1989; Olff & Ritchie, 1998). Recent studies have improved understanding of how herbivory affects aggregate metrics such as species diversity and overall biomass, as well as how plant traits and environmental context modify herbivore impacts (Borer et al., 2014; du Toit & Olff, 2014; Endara & Coley, 2011; Jia et al., 2018; Karp et al., 2024; Koerner et al., 2018; Price et al., 2022; Staver et al., 2021; Trepel et al., 2024). But it has remained unclear how well herbivore selectivity predictably translates into community‐level impact and whether it is an essential predictor or instead redundant with plant traits that are easier to measure.

Our primary finding is that the foraging selectivity of elephants predicted the responses of plants to a decade of large‐herbivore exclusion, explaining between a quarter and a third of the variance and outperforming all other predictors. Although elephant selectivity predicted the magnitude of plant responses equally well in all three exclosure treatments relative to control (unfenced) plots, we did not detect a meaningful signal of any herbivore species in contrasts between successive exclosure treatments. The plant traits that we analyzed were able to explain an appreciable proportion of the variation in herbivore selectivity, notably for the largest and smallest grazers and browsers. Collectively, these traits enhanced predictions of plant community response relative to elephant selectivity alone, but they did not substitute for it.

### Impacts of herbivore exclusion: Primacy of megaherbivores and indirect effects

Our finding of a strong effect of elephant foraging activity is in keeping with previous studies, including several in UHURU (Coverdale et al., 2016, 2021, 2024; Pringle et al., 2014), showing that elephants drastically affect plant vital rates and vegetation structure (Abraham et al., 2021; Davies et al., 2018; Guldemond et al., 2017; Laws, 1970). In part, elephants' impact arises from the energetic requirements associated with huge body size—other megaherbivores, such as white rhino (Cromsigt & te Beest, 2014) and hippo (Voysey et al., 2023), can be similarly influential where they occur at high densities—and elephant biomass at Mpala is higher than that of any other species (on the order of 3000 kg km^−2^; Augustine, 2010). Yet, our results also show that selective foraging by elephants for particular plant taxa is crucial in regulating community composition. All else equal, big herbivores are expected to be unselective feeders, in part due to morphological constraints and in part due to their high intake requirements (Clauss et al., 2013; Owen‐Smith, 1988); in principle, unselective foraging should tend to homogenize impacts across plant taxa (Augustine & McNaughton, 1998). But elephants are uniquely able to bulldoze trees while also foraging nimbly with their trunks, which enables them to be simultaneously disruptive and selective (Abraham et al., 2021). Indeed, elephants have higher quality diets than predicted by allometric regressions (Clauss et al., 2013; Owen‐Smith, 1988; Potter et al., 2022), and they use nuanced olfactory cues to guide their discriminating feeding decisions (Schmitt et al., 2018).

We expected to find some evidence for an additional effect of smaller herbivores, which strongly influence understory biomass and diversity in the UHURU exclosures (Coverdale et al., 2024). Dik‐dik in particular reach high densities (up to 140 individuals and 700 kg km^−2^) and are highly selective in ways that curb plant recruitment (Augustine & Mcnaughton, 2004). Also, dik‐dik and impala diets are more diverse than, and compositionally distinct from, elephant diets (Kartzinel et al., 2015; Kartzinel & Pringle, 2020). We had thus predicted that the best models for MESO and TOTAL treatments would include dik‐dik, impala, or both. They did not, in part because many of the plants selected by elephants exhibited similar responses in all of three of the exclosure treatments (Figure 3), and in part because these species' selectivities were positively correlated, making them somewhat redundant as predictors (Appendix S1: Figure S5).

These factors contextualize the failure of herbivore selectivity to predict differences between exclosure treatments. Megaherbivore exclusion triggered shifts in plant composition that were stable regardless of which other herbivore species were excluded. These shifts were defined largely by increases in woody plants and vines that were positively selected by most or all of the browsing herbivore species. For example, the shrubs *Senegalia brevispica* (*D*_ele_ = 0.82, *D*_gir_ = 0.78, *D*_imp_ = 0.66, *D*_dik_ = 0.60) and *Grewia* spp. (*D*_ele_ = 0.62, *D*_gir_ = 0.24, *D*_imp_ = 0.40, *D*_dik_ = 0.58) were each tenfold more abundant in MEGA than OPEN controls but differed little among MEGA, MESO, and TOTAL. In contrast, the further shifts that occurred between this megaherbivore‐free “baseline” and the exclosures treatments targeting smaller herbivores were defined less by universally selected shrubs and more by an eclectic set of grasses and forbs. Grasses such as *Digitaria* (selected only by zebra and buffalo) and *Pennisetum* spp. (avoided), along with forbs such as *Ipomoea* (avoided), *Indigofera* (moderately selected), and *Commelina* (ranging from avoided to selected) were all several‐fold more common in MESO plots relative to MEGA plots, and/or in TOTAL plots relative to MESO plots. An equally variable set of taxa, including grasses (e.g., *Aristida adscensionis*, avoided except by zebra), forbs (e.g., *Pavonia* spp., selected except by zebra; *Ruellia* spp., selected only by impala and dik‐dik), and subshrubs (e.g., *Barleria spinisepala*, avoided) declined sharply. Collectively, the tendency of (a) overstory taxa to persist, (b) herbivore‐avoided understory taxa to increase, and (c) herbivore‐selected understory taxa to decline are consistent with a scenario in which consumptive (herbivore–plant) effects attenuate relative to competitive (plant–plant) effects as herbivores are lost (Coverdale et al., 2016, 2024; Louthan et al., 2014).

### Plant traits as predictors of herbivore diet and plant response

The traits predictive of herbivore selectivity were not the ones we had anticipated, but nonetheless a limited number of traits predicted herbivore selectivity to a substantial degree. Our efforts to predict selectivity using combinations of plant traits were most successful for the biggest, smallest, and most specialized herbivores, and least successful for the two midsized mixed feeders, impala and buffalo (Figure 2).

Despite this variability, most herbivores were correlated in their patterns of selectivity (Appendix S1: Figure S5), and several plant traits were consistently predictive of selectivity for multiple herbivore species. We had hypothesized that the traits most predictive of selectivity would be those canonically associated with plant nutritional quality (e.g., protein, digestibility), herbivore mineral nutrient deficiencies (e.g., Na), and antiherbivore resistance (e.g., spines, leaf toughness) (Augustine & McNaughton, 1998). Several of these traits were indeed predictive for some herbivores, but not with the expected relative importance or even direction (Figure 2). For example, spiny plants were avoided by most herbivores, as predicted, but Cu was avoided as well, with a strength and consistency that we are presently unable to explain. The distribution of Cu concentrations across taxa ranged from 2 to 22 ppm, and plants at the high end of that range were predominantly grasses (including several that are usually considered unpalatable, such as *Heteropogon* and *Bothriochloa* spp. but also conventionally palatable taxa, such as *Digitaria*), along with alkaloid‐rich Solanaceae (*Solanum*, *Datura*), and aromatic Lamiaceae (*Plectranthus*, *Ocimum*, *Endostemon*).

Protein, thought to be a key driver of selectivity for small herbivore species especially (Bell, 1971; Clauss et al., 2013; Jarman, 1974; Olff et al., 2002; Potter et al., 2022), was indeed a strongly positive predictor of dik‐dik but also elephant selectivity (the smallest and largest herbivores analyzed here) and was marginally predictive for impala; protein was a strong negative predictor of zebra selectivity among grasses, reflecting the tendency of zebra at this site to actively select protein‐poor species such as *Brachiaria*, *Aristida*, and *Setaria* (notably, species that exhibited low leaf toughness and Zn concentrations, which ultimately best predicted zebra selectivity). Nonetheless, despite uncertainty about the biological mechanisms by which these plant traits govern selectivity, top models of herbivore selectivity generally included only 2–5 plant traits, and never more than 7 (Appendix S1: Table S2). That (a) patterns of selectivity were correlated across herbivore species, (b) plant traits were predictive for multiple herbivores, and (c) selectivity was predictable from so few traits implies that a low‐dimensional set of axes might suffice to characterize dietary differences between herbivore species (Potter et al., 2022).

Yet, although plant traits predicted herbivore selectivity, they were unable to substitute for elephant selectivity in predicting plant responses to exclusion. If traits outperformed or substituted for elephant selectivity, then our model selection should have revealed that. To the contrary, elephant selectivity was retained in all supported models, even when plant traits were also included (Appendix S1: Tables S3 and S4). It is possible that we did not measure the most relevant traits: perhaps traits more proximately linked with tolerance and/or competition, such as photosynthetic rate, tillering, or root:shoot ratio, might have superseded herbivore selectivity (Augustine & McNaughton, 1998; Milchunas et al., 1988; Noy‐Meir et al., 1989; Olff & Ritchie, 1998; Strauss & Agrawal, 1999). However, it is difficult to identify which traits are most relevant to predicting outcomes of species interactions; community ecologists have for decades sought to predict such outcomes from plant traits, with mixed success (Anderegg, 2023; Kraft et al., 2015; Levine, 2016; Levine et al., 2024; Streit & Bellwood, 2023). Recent work has increasingly emphasized the importance of computing metrics that capture key elements of the underlying mechanistic processes at work, which often requires combining multiple functional traits and/or demographic characteristics (Kraft et al., 2015; Levine et al., 2024). In the case of herbivore–plant interactions, herbivore choosiness regarding which plants to eat is obviously relevant, and our study suggests that it is an essential component to measure.

### Towards a predictive empirical framework: Caveats and future directions

We devised our approach in full awareness of its limitations. These include that metabarcoding data imperfectly quantifies consumption (Deagle et al., 2019; Littleford‐Colquhoun et al., 2022); that potentially relevant plant traits were not measured; that our estimates of plant abundance were approximate and only indirectly calibrated; that predictive correlative models cannot presume to reveal underlying mechanisms (Levine et al., 2024; Maris et al., 2018); and that compounding measurement errors at each stage in the analytical pipeline could potentially drown out biologically meaningful signals. We accepted these risks, motivated by the need to develop a practical and generalizable framework for problems that otherwise are simply neglected.

Our approach presupposes that underlying mechanistic relationships among herbivory selectivity, plant traits, and responses to herbivore exclusion translate into statistical associations that are strong enough to be detectable despite these limitations and through various sources of noise, including measurement error, spatial and intraspecific variation, and our proxy measurement of selectivity. In general and to varying degrees, they did: we found elephant selectivity to be a crucial predictor of plant responses to herbivore removal. That *D*_ele_ predicted responses as well as it did is impressive, but it still left most variance unexplained. Several factors likely contribute to this unexplained variance, which we stress here. First, quantitative interpretation of relative abundance in metabarcoding data is necessary to estimate feeding selectivity but is at best an approximate indicator of true intake (Deagle et al., 2019), just as any sampling of plant relative abundance can only approximate the landscape of “availability” to herbivores. All methods of estimating selectivity are imperfect, but ours has the advantage of being estimable with taxonomic scope and precision that is otherwise effectively unattainable. However, we acknowledge the likelihood that noise in our estimates of selectivity reduced the fidelity of our predictions and our ability to detect impacts of smaller ungulates. Along similar lines, selectivity measures choice, not consumption: a plant that is avoided relative to its abundance may still be eaten in large quantities. There is no doubt that consumption by smaller ungulates has shaped the differentiation of plant communities across the UHURU treatments, and it is possible that more direct estimation of offtake (minimally requiring better data on herbivore densities) would predict these effects (Karp et al., 2024; Staver et al., 2021). Selectivity also does not measure pure preference—it changes when the food availability changes (Abraham, Rowan, et al., 2024; Jacobs, 1974; Wam & Hjeljord, 2010)—such that our estimates (which are based on average patterns of consumption and availability when all herbivores are present) may not reflect choices made in plots where megaherbivore exclusion has already modified what is available.

While we find that plant traits explain variation not accounted for by elephant selectivity, we also do not know to what extent these trait effects reflect plant tolerance to herbivory, are pivotal to competitive dynamics, or (likely) both. Future work that combines experimental approaches, long‐term demographic data, and additional traits that have been more explicitly linked to variation in tolerance and competitive ability could explore some puzzling results that have emerged from this study and thus help to clarify the mechanistic bases and relative importance of these pathways.

Limitations notwithstanding, our study makes clear that information about herbivore foraging decisions is necessary for predicting plant responses to herbivory—and indeed sufficient for a first‐order characterization of both community‐level vegetation change and the pivotal role of an endangered keystone megaherbivore. Data on plant traits did not substitute for data on herbivore–plant interactions but did complement them. Our findings are a hopeful step in the quest for predictability in the ecology of complex natural systems, and we expect that future work can refine our approach to achieve even more precise and accurate predictions.

## AUTHOR CONTRIBUTIONS

Joel O. Abraham and Robert M. Pringle conceived and designed the study. Jacob R. Goheen, Todd M. Palmer, and Robert M. Pringle conceived and coordinated the experiment. Samson Kurukura and Abdikadir Ali Hassan maintained the experiment. Maria Stahl, Samson Kurukura, Abdikadir Ali Hassan, Tyler R. Kartzinel, and Robert M. Pringle collected data. Joel O. Abraham and Robert M. Pringle analyzed data and wrote the manuscript. All coauthors read and approved the final manuscript.

## CONFLICT OF INTEREST STATEMENT

The authors declare no conflicts of interest.

## Supporting information


Appendix S1:

Appendix S2:

## Data Availability

Raw data and R scripts (Abraham et al., 2026) are available in Dryad at https://doi.org/10.5061/dryad.41ns1rntq.
