# Supplementary material for: Impacts of large herbivores on savanna plant communities: Predictive models of herbivore selectivity and plant response
Source: Ecology. 2026 Jul 23;107(7):e70445. doi: 10.1002/ecy.70445 (PMC13394317; doi:10.1002/ecy.70445)
Supplement: Supplementary file 1 — Appendix S1: [file ECY-107-e70445-s002.pdf]

APPENDIX S1

Impacts of large herbivores on savanna plant communities: Predictive models of herbivore selectivity and plant response

Joel O. Abraham, Maria Stahl, Samson Kurukura, Abdikadir Ali Hassan, Jacob R. Goheen, Todd M. Palmer, Tyler R. Kartzinel, Robert M. Pringle

Ecology

This Appendix contains:

Figures S1–S9
Tables S1–S8

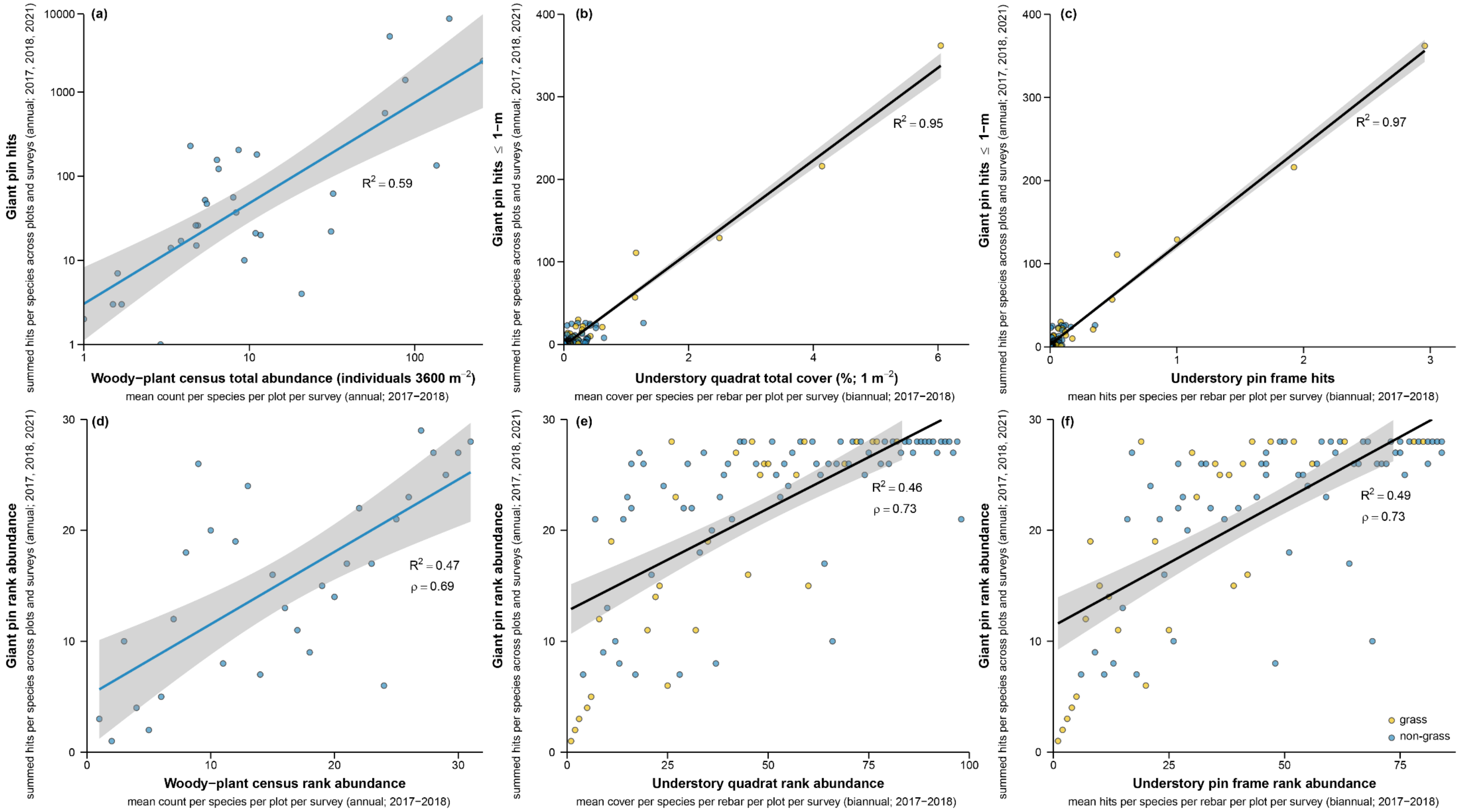

Figure S1. Validation of experimental plant-response estimates based on modified canopy-intercept ('giant pin') survey method. Total giant-pin hits per plant species in exclosure plots (summed across plots and surveys) were highly correlated with contemporaneous measurements in the same plots using conventional survey methods. **(a–c)** Relative abundance in our data was highly correlated (R^2 = 0.59–0.97) with: (a) woody-plant density (averaged across plots and surveys, note log-log axes); (b) percent cover of understory species in 1-m^2 quadrats (averaged across quadrats, plots, and surveys); and (c) density of understory species measured with a 10-pin frame in each quadrat (averaged across all frame placements, plots, and surveys). **(d–f)** Rank abundances of species in the same datasets were also well-correlated (Spearman's rank correlation coefficient, ρ = 0.69–0.73). Plant species are colored by plant functional type (yellow, grasses; blue, non-grasses). R^2 values are from linear regressions (fits and shaded 95% CI). Saturating shapes in (e) and (f) occur in part because species with equal abundance were assigned equal ranks, and many rare species were recorded fewer than four times across all surveys.

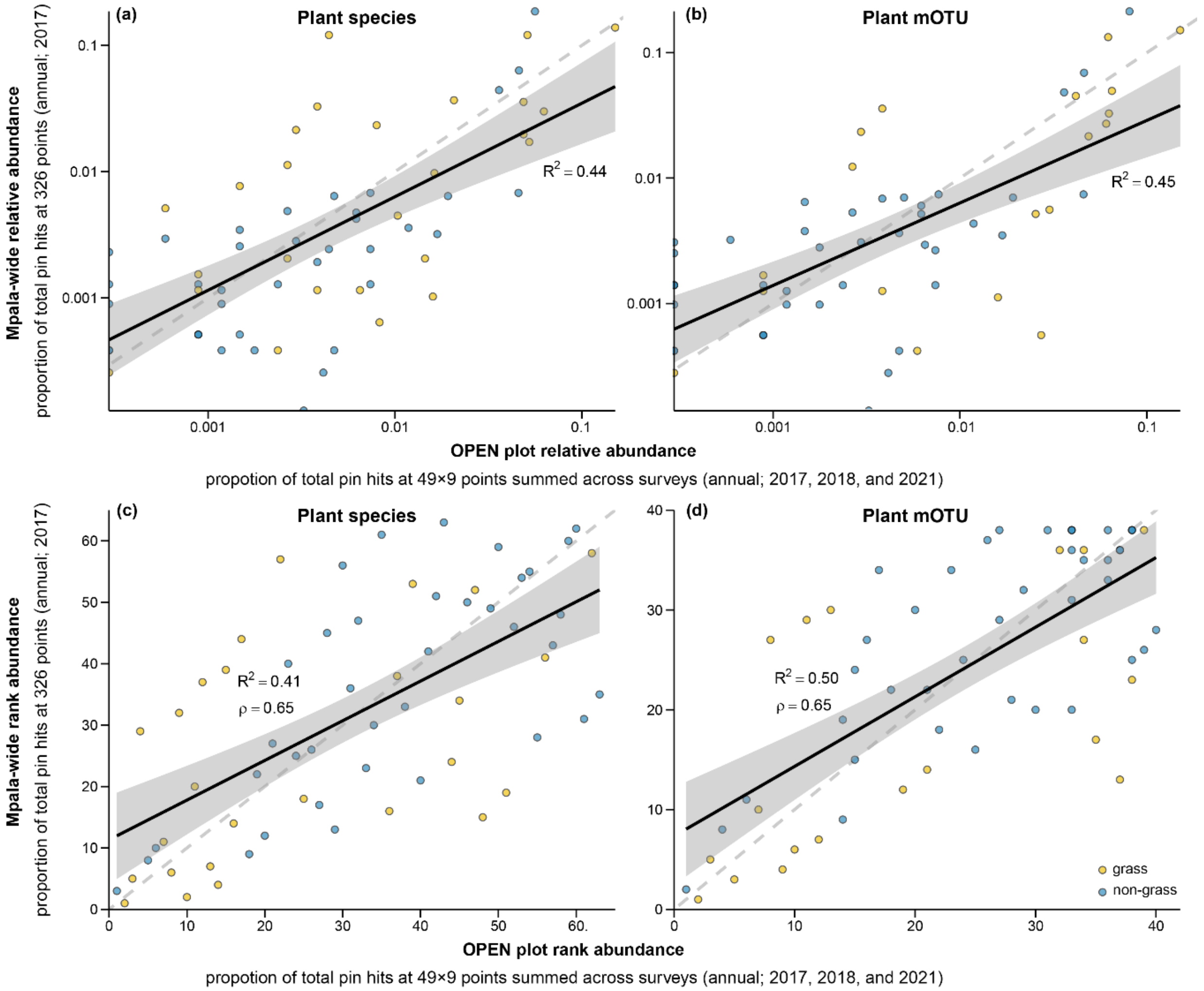

Figure S2. Validation of relative-abundance estimates from the plant-availability survey. Data from our Mpala-wide survey of plant relative abundance (N = 326 giant-pin placements), used to estimate herbivore selectivity, were well-correlated with data from more intensive (but more spatially clustered) sampling using the same giant-pin method in unfenced (OPEN) UHURU plots. **(a, b)** Proportional relative abundance, R^2 = 0.44–0.45 (note log–log axes). **(c, d)** Rank abundance, Spearman's rank correlation coefficient, $\rho = 0.65$. Points represent plant taxa, colored by plant functional type (yellow, grasses; blue, non-grasses). In the panels at left, points are individual species, as originally measured; at right, taxonomy is coarsened to mOTUs (one or more species), for correspondence with the dietary data. R^2 values are from simple linear regression (fitted line and shaded 95% CI). Dashed gray line shows 1:1 correspondence.

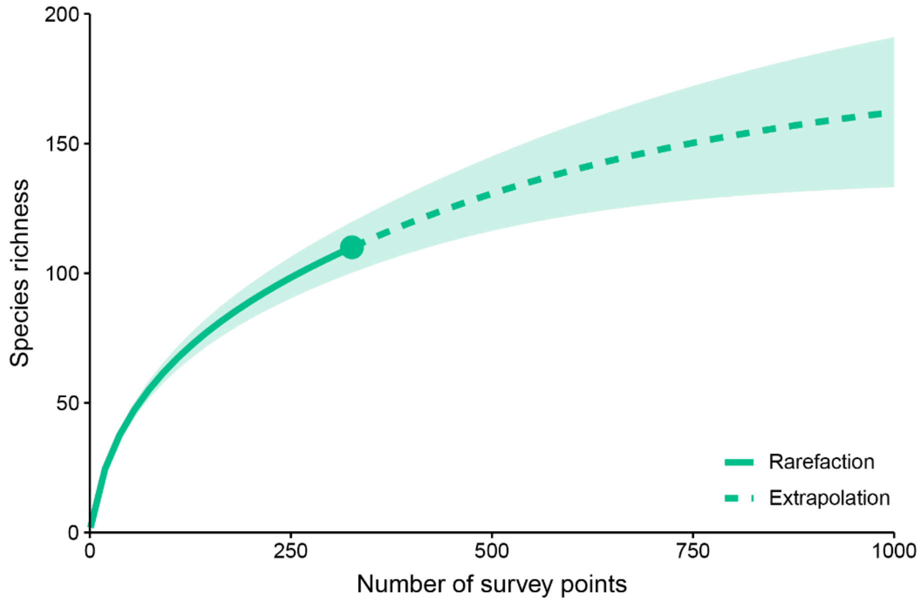

Figure S3. Plant species accumulation as a function of sampling effort in the availability survey. The species accumulation curve indicates that our sampling effort was sufficient to capture all but the rarest species, which are unlikely to be major components of herbivore diets (see **Fig. S4**), and that even substantially greater (*e.g.,* doubled) effort would not massively increase overall species coverage. Point corresponds to realized sampling effort (326 survey points) and observed species richness (109 species). Solid line shows sample-based rarefaction within the range of the data; dashed line shows extrapolated estimates beyond the range of the data, up to 1,000 survey points. Shaded regions show the 95% confidence interval around the species accumulation curve.

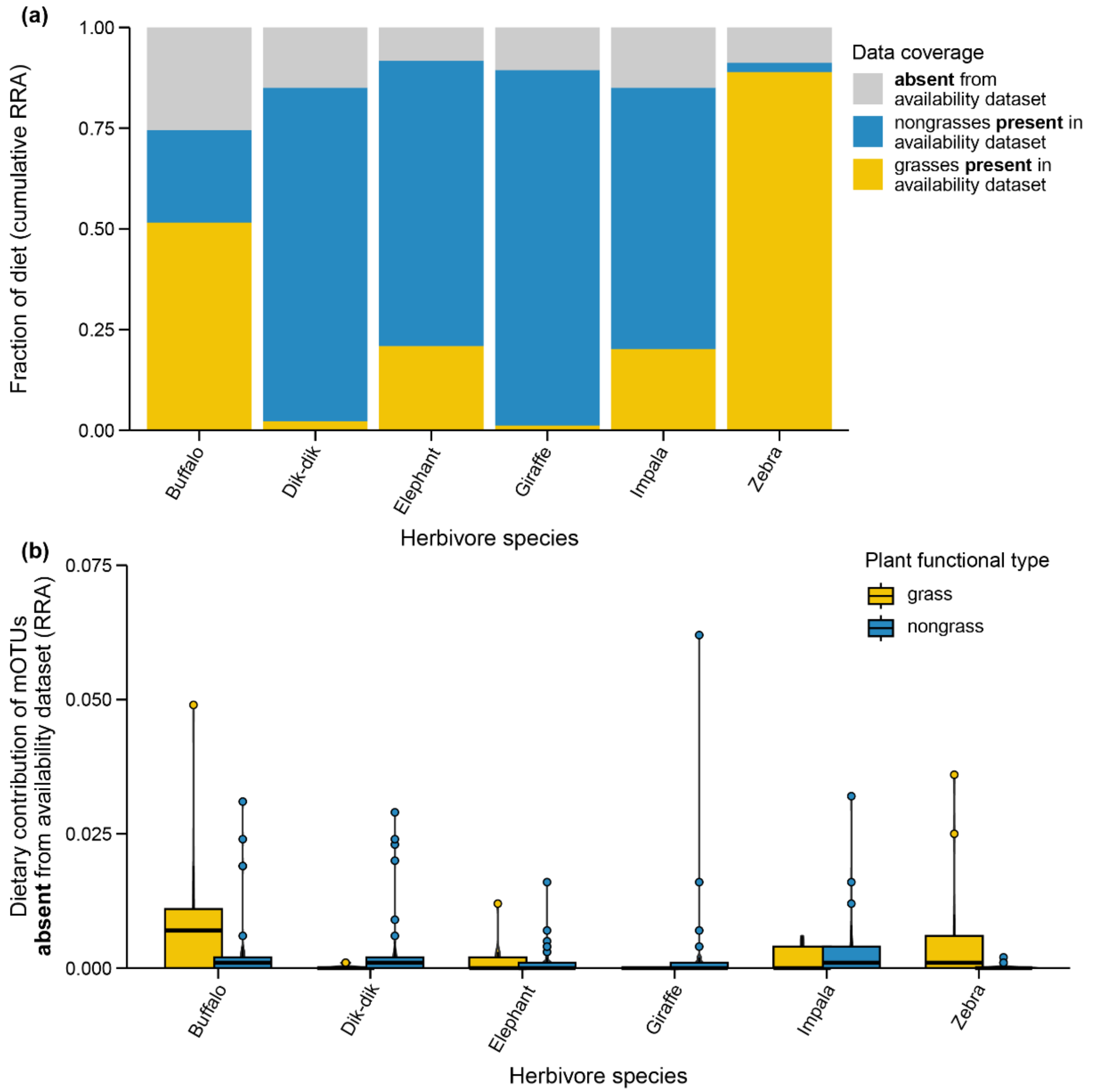

Figure S4. Plant taxa detected in the availability survey as a proportion of herbivore diets. Plant taxa that were not detected in the availability survey, and that were therefore excluded from herbivore selectivity estimates, made up a small minority of herbivores' average diet profiles. **(a)** For each herbivore species, stacked bars show proportion of the diet (relative read abundance, RRA) consisting of plant mOTUs for which at least one constituent species was detected in the availability survey (colored) versus those for which none of the constituent species was detected (gray: range 8–26%, median 13%). **(b)** Mean RRA of individual plant mOTUs that were absent from the availability data (at most 6.2% of any herbivore species' diet). Boxes represent the interquartile range (IQR), center lines show median, whiskers span the range, and dots are outliers. For each herbivore species, the summed RRA of all mOTUs depicted in (b) is equivalent to the gray fraction of the stacked bars in (a). Colors indicate plant functional type (yellow, grasses; blue, non-grasses).

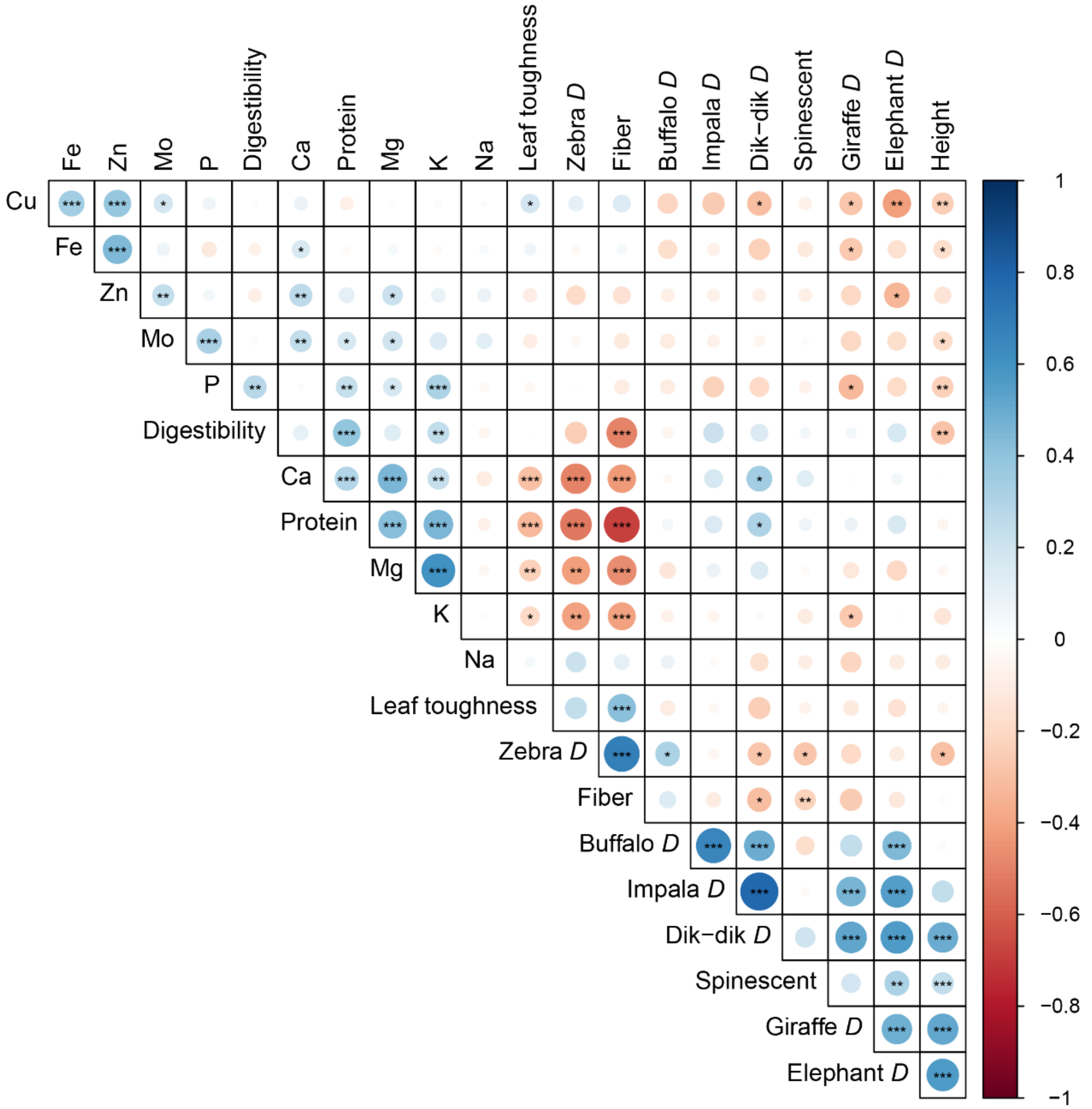

Figure S5. Pairwise correlations between plant traits and herbivore selectivity estimates (*D*). Circle colors and sizes show direction and magnitude of each correlation (Pearson's r), as per the color gradient at right. Asterisks indicate statistical significance at different thresholds ($^{*}\ P < 0.05$; $^{**}\ P < 0.01$; $^{***}\ P < 0.001$). Variables are ordered by similarity, according to hierarchical clustering. Pairs of highly correlated traits ($r > 0.7$) were not included together as predictors in the same candidate multiple-regression model.

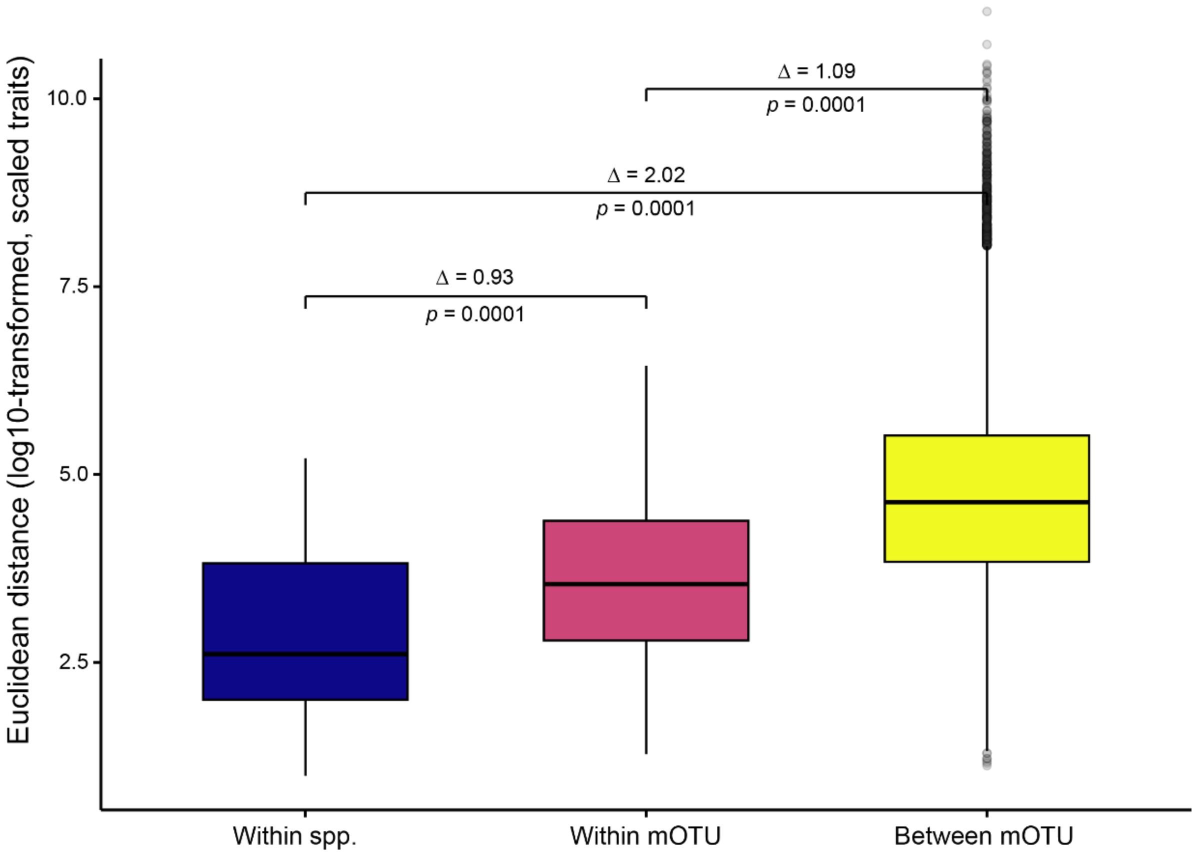

Figure S6. Similarity of plant traits within and between mOTUs. Many plant mOTUs in the herbivore diets match multiple plant species. As species within mOTUs are generally closely related (same genus or family), we assumed that they have similar trait values and thus averaged species' trait data within mOTUs to predict herbivore selectivity. We validated this assumption by calculating and comparing Euclidean distances between trait values (a) in different samples of the same species (blue, reflecting repeatability of intraspecific measurements); (b) between species in the same mOTU (pink); and (c) between species in different mOTUs (yellow). Boxes represent the interquartile range (IQR), center lines show median, whiskers span 1.5×IQR, and dots are outliers. We used the set of complete mineral-nutrient data (ppm of Fe, Cu, Zn, Mn, Na, B, and Mo; percent of S, Mg, K, P, Ca) for 224 plant species, which include data from up to 3 replicate samples per species ($N = 260$ individuals); data were $\log_{10}$-transformed and standardized (centered and scaled) prior to calculating Euclidean distances. We calculated the difference (Δ) between the medians of each pair and tested the statistical improbability of those differences being random using permutation tests, in which we randomly reassigned species to mOTUs but preserved the number of lumped species per mOTU (10,000 iterations; $P = 0.0001$ for all three comparisons). As expected, distances were lowest within species and lower within mOTUs than between them. Whereas the IQR of within-mOTU distances largely overlapped the IQR of within-species distances (*i.e.,* trait variation between species within mOTUs is often indistinguishable from trait variation within species), the IQR of between-mOTU distances was uniformly higher (*i.e.,* species lumped within the same mOTU had more similar traits than species in different mOTUs). We conclude that mOTUs are coherent in terms of traits, validating our original assumption.

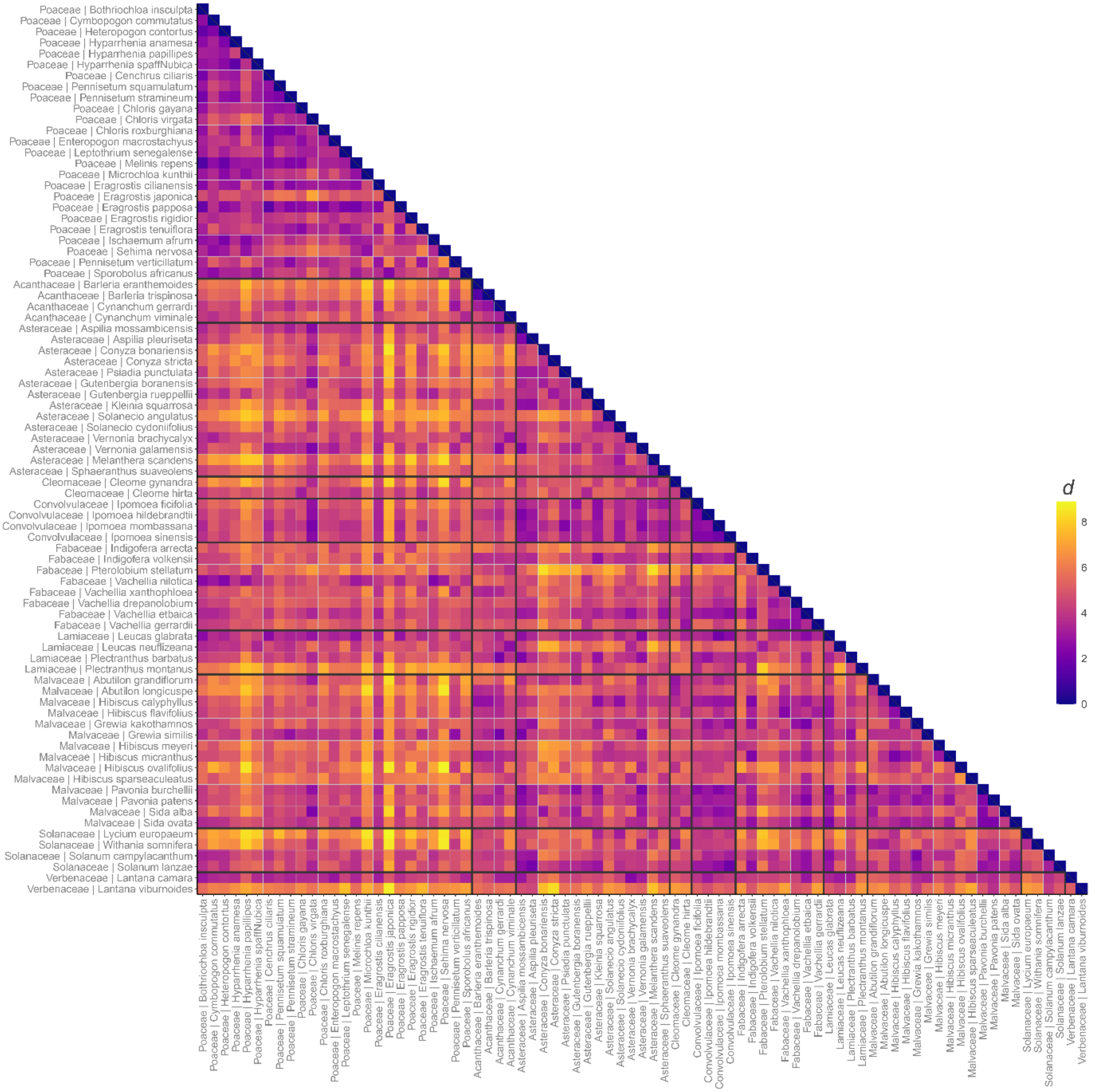

Figure S7. Visualizing the matrix of pairwise Euclidean distances (*d*) between plant species in standardized mineral-nutrient trait space. We calculated *d* based on foliar mineral-nutrient data (ppm of Fe, Cu, Zn, Mn, Na, B, and Mo; percent of S, Mg, K, P, Ca) between pairs of species lumped in multi-species mOTUs. Species are ordered first by family (families are delineated by heavier black lines) and then by mOTU identity (delineated by thinner light lines). More purple colors indicate trait similarity (lower *d*); yellower colors indicate divergence (high *d*). Triangular arrays closest to the diagonal—contrasts between species belonging to the same family and mOTU—exhibit some of the lowest *d* values, indicating generally higher trait similarity within taxonomic units than between them (**Fig. S6**). For instance, the topmost triangle shows high similarity within an mOTU comprising five panicoid grasses (*Cymbopogon*, *Heteropogon*, and *Hyparrhenia* spp.).

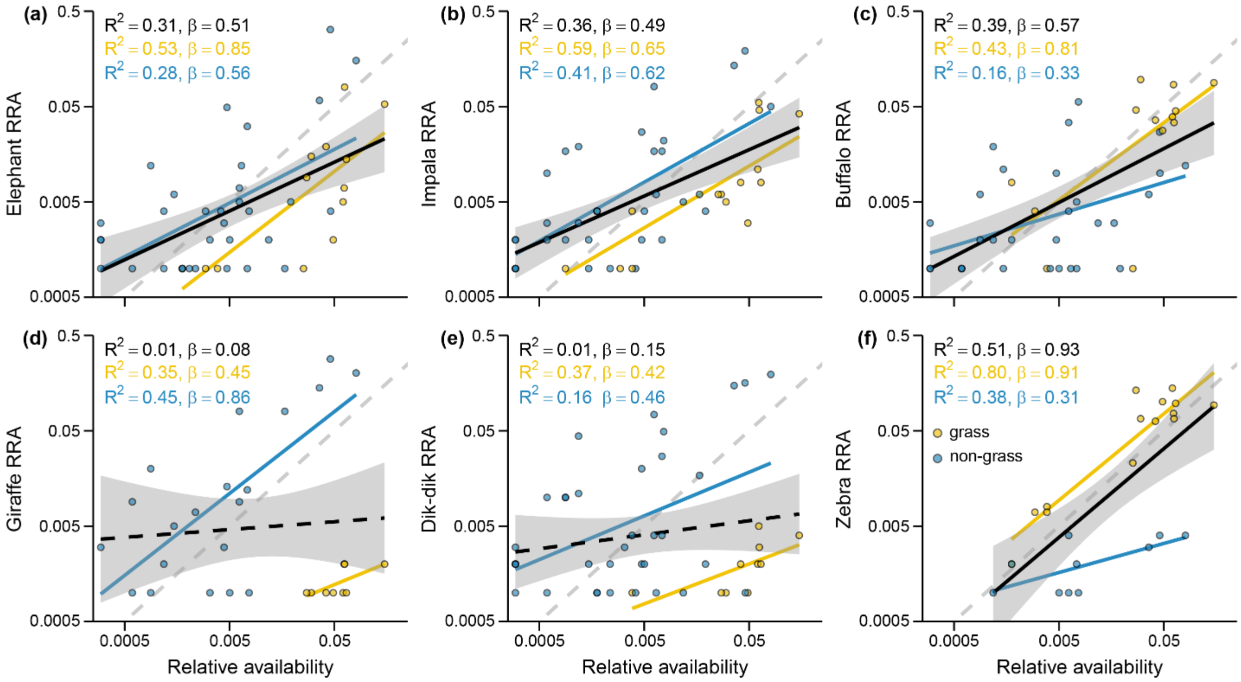

Figure S8. Plant relative availability predicts mean relative read abundance. Plant taxa that were more abundant in the landscape were also more heavily represented in herbivore species' average diet profiles. Plant mOTUs are colored by plant functional type (yellow, grasses; blue, non-grasses). Black lines and shaded 95% CI show overall linear regression fits including all mOTUs (dashed where the overall relationship is statistically equivocal, $P > 0.05$); yellow and blue lines show fits for grasses only and non-grasses only, respectively. Corresponding R^2 values and slopes are in the top left. Dashed gray lines show 1:1 correspondence. All axes are log-transformed.

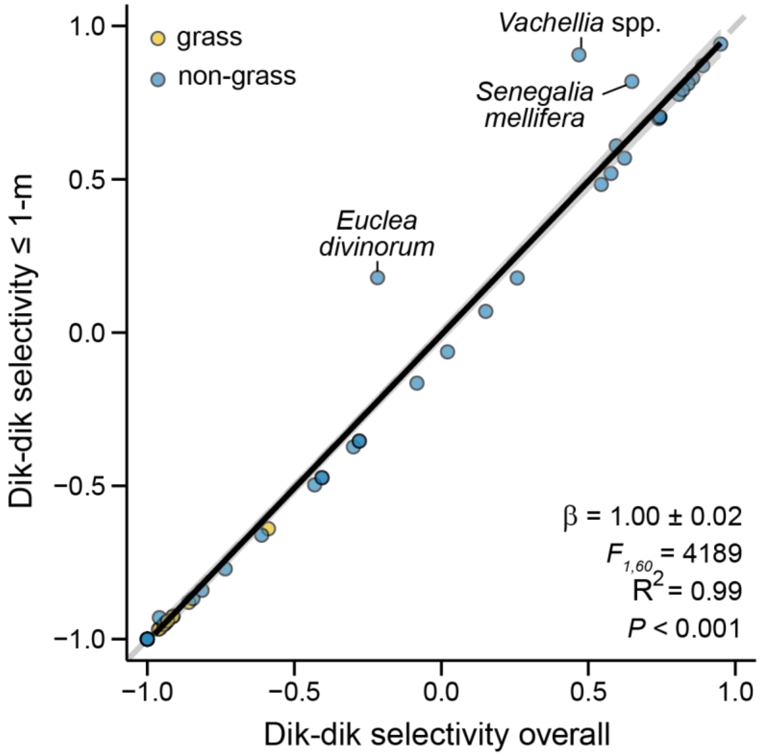

Figure S9. Comparing two methods of quantifying dik-dik foraging selectivity. Selectivity values calculated using the complete plant relative-availability dataset (*x*-axis) were practically indistinguishable from those in which relative availability was estimated only for vegetation ≤ 1-m high (*i.e.*, the subset of forge that dik-dik can reach; *y*-axis), with slope = 1.00 and R^2 = 0.99. The only deviations involved three abundant woody taxa, the acacias *Senegalia mellifera* and *Vachellia* spp. (including *V. etbaica*, one of the locally dominant species) along with the shrub *Euclea divinorum*, for which selectivity was greater using vertically restricted availability data. Point color corresponds to plant functional type (yellow, grasses; blue, non-grasses).

Table S1. Selectivity estimates. The selectivity of each herbivore species for each plant taxon was calculated as $D = (d - a) / (d + a - 2da)$, where d is the proportion of the mOTU in the diet and a is its proportion in the environment. D ranges from -1 to 1; positive values indicate selection (more common in diet than environment) and negative values indicate avoidance (relative to availability). We assumed d equal to the relative read abundance (RRA) of the mOTU in the mean diet for each herbivore species (averaging across individuals, time, and space) and a equal to the mean relative abundance of all plant species subsumed within the mOTU. In addition to calculating D based on overall relative availability, we calculated grass-specific (for zebra), browse-specific (for giraffe and dik-dik), and height-specific (for dik-dik) variants, as described in the main text.

Seq. ID	Family	Potential species	Elephant D_{ele}	Impala D_{imp}	Buffalo D_{buf}	Zebra D_{zeb}	Zebra, grass $D_{\mathrm{zeb\ grass}}$	Giraffe D_{gir}	Giraffe, non-grass $D_{\mathrm{gir\ non}}$	Dik-dik D_{dik}	Dik-dik, non-grass $D_{\mathrm{dik\ non}}$	Dik-dik, ≤1-m $D_{\mathrm{dik} \leq 1\mathrm{m}}$
seq_ 000002	Fabaceae	*Indigofera volkensii; Indigofera arrecta; Indigofera circinella; Indigofera bogdanii*	-0.108	0.868	0.699	-0.217	NA	0.185	-0.305	0.855	0.648	0.831
seq_ 000003	Poaceae	*Panicum maximum; Urochloa brachyura; Brachiaria lachnantha; Brachiaria semiundulata; Eriochloa fatmensis*	-0.342	-0.721	0.547	0.396	0.164	-0.938	NA	-0.938	NA	-0.947
seq_ 000004	Poaceae	*Digitaria macroblephara; Digitaria velutina*	-0.807	-0.064	0.168	0.109	-0.155	-0.941	NA	-0.859	NA	-0.880
seq_ 000010	Fabaceae	*Senegalia brevispica*	0.816	0.664	-0.267	-0.846	NA	0.783	0.473	0.595	0.145	0.609
seq_ 000011	Poaceae	*Pennisetum stramineum; Pennisetum squamulatum*	-0.52	-0.604	-0.29	-0.267	-0.52	-0.978	NA	-0.956	NA	-0.964
seq_ 000012	Poaceae	*Cymbopogon commutatus; Hyparrhenia anamesa; Hyparrhenia papillipes; Hyparrhenia filipendula; Bothriochloa insculpta; Hyparrhenia sp. aff. Nubica*	-0.855	-0.706	-0.227	0.433	0.196	-0.969	NA	-0.94	NA	-0.949
seq_ 000013	Fabaceae	*Vachellia reficiens; Vachellia etbaica; Vachellia gerrardii; Vachellia tortilis; Vachellia drepanolobium*	0.343	-0.25	-0.757	-0.912	NA	0.485	-0.048	0.468	-0.07	0.906
seq_ 000017	Poaceae	*Melinis repens; Tetrapogon cenchroides; Microchloa kunthii; Leptothrium senegalense; Leptochloa obtusiflora; Enteropogon macrostachyus; Chloris roxburghiana*	-0.383	-0.687	-0.076	0.215	-0.039	-0.955	NA	-0.912	NA	-0.925
seq_ 000018	Fabaceae	*Senegalia mellifera*	0.245	0.614	-0.722	-0.851	NA	0.629	0.207	0.648	0.238	0.819
seq_ 000021	Rubiaceae	*Pentanisia ouranogyne*	-1	0.902	0.744	-1	NA	0.061	-0.411	0.839	0.616	0.812
seq_ 000028	Poaceae	*Cynodon nlemfuensis; Eustachys paspaloides*	0.131	-0.162	-0.31	0.036	-0.225	-0.942	NA	-0.914	NA	-0.927
seq_ 000029	Phyllanthaceae	*Phyllanthus sepialis; Phyllanthus rotundifolius; Phyllanthus suffrutescens*	0.831	0.708	0.361	-1	NA	0.47	0.009	0.623	0.224	0.569
seq_ 000033	Poaceae	*Themeda triandra*	-0.925	-0.889	-0.28	0.374	0.132	-0.962	NA	-0.962	NA	-0.968
seq_ 000035	Malvaceae	*Pavonia*	0.606	0.151	0.765	-1	NA	0.545	0.113	0.151	-0.333	0.069

seq_ 000037	Caryophyllaceae	*Pollichia campestris*	-0.763	-0.576	-0.763	-1	NA	-1	-1	-0.299	-0.67	-0.373
seq_ 000041	Phyllanthaceae	*Phyllanthus maderaspatensis*	-0.083	0.885	0.885	-0.083	NA	0.258	-0.23	0.95	0.87	0.941
seq_ 000046	Acanthaceae	*Ruellia prostrata; Ruellia patula; Ruellia sp.*	-1	0.743	-1	-1	NA	-1	-1	0.544	0.112	0.483
seq_ 000056	Poaceae	*Dactyloctenium aegyptium*	-0.279	0.387	0.639	0.061	-0.182	-1	NA	-1	NA	-1
seq_ 000057	Asteraceae	*Vernonia cierascens; Vernonia galamensis; Vernonia brachycalyx; Solanecio cydoniifolius; Solanecio angulatus; Gutenbergia rueppellii; Gutenbergia boranensis; Senecio hadiensis; Galinsoga parviflora; Kleinia squarrosa; Schkuhria pinnata*	-0.652	-0.406	-0.406	-1	NA	-1	-1	-0.406	-0.732	-0.474
seq_ 000059	Poaceae	*Pennisetum mezianum; Cenchrus ciliaris*	-0.661	-0.792	-0.192	0.214	-0.048	-0.972	NA	-0.944	NA	-0.953
seq_ 000062	Commelinaceae	*Commelina reptans; Commelina africana*	-0.713	-0.408	-0.598	-1	NA	-1	-1	-0.845	-0.941	-0.868
seq_ 000067	Poaceae	*Eragrostis superba*	-0.495	-0.495	0.151	0.408	0.186	-1	NA	-1	NA	-1
seq_ 000068	Ebenaceae	*Euclea divinorum*	0.061	0.47	-0.217	-0.724	NA	0.866	0.672	-0.217	-0.619	0.179
seq_ 000070	Commelinaceae	*Commelina erecta*	0.743	0.544	0.544	-1	NA	-1	-1	0.743	0.43	0.703
seq_ 000073	Euphorbiaceae	*Croton*	-0.846	-1	-0.654	-1	NA	-1	-1	-0.959	-0.986	-0.930
seq_ 000075	Poaceae	*Brachiaria eruciformis; Chrysopogon plumosus; Melinis repens; Panicoideae sp; Setaria pumila; Setaria sphacelata; Setaria verticillata; Sporobolus africanus; Sporobolus discosporus; Sporobolus festivus; Sporobolus stapfianus; Sporobolus sp.*	-0.509	-0.645	0.266	0.692	0.536	-0.931	NA	-0.931	NA	-0.941
seq_ 000077	Malvaceae	*Hibiscus calyphyllus; Malva parviflora; Hibiscus micranthus; Abutilon sp.; Hibiscus ovalifolius*	-0.317	0.488	0.769	-0.771	NA	-0.771	-0.91	0.739	0.417	0.698
seq_ 000084	Malvaceae	*Grewia similis; Grewia kakothamnos*	0.623	0.398	-0.194	-0.576	NA	0.24	-0.253	0.577	0.154	0.519
seq_ 000085	Convolvulaceae	*Ipomoea ficifolia; Ipomoea hildebrandtii; Ipomoea mombassana; Ipomoea sinensis*	0.545	0.436	0.258	-1	NA	-1	-1	0.808	0.553	0.777
seq_ 000095	Solanaceae	*Solanum coagulans; Solanum polhillii; Solanum lanzae; Solanum campylacanthum; Solanum taitense; Solanum tettense*	0.3	-0.04	-0.735	-1	NA	-1	-1	-0.735	-0.894	-0.771
seq_ 000123	Poaceae	*Eragrostis japonica; Eragrostis heteromera; Eragrostis cilianensis; Eragrostis racemosa; Eragrostis tenuiflora; Eragrostis papposa;*	-0.926	-0.624	-0.926	-0.051	-0.294	-1	NA	-1	NA	-1

		Eragrostis braunii; Eragrostis cylindriflora; Eragrostis sp.									
seq_ 000126	Acanthaceae	*Justicia debilis*	-1	0.672	0.258	-1	NA	0.258	-0.23	0.258	-0.23	0.178
seq_ 000142	Poaceae	*Harpachne schimperi*	-0.588	0.021	-0.588	0.353	0.123	-1	NA	-0.588	NA	-0.64
seq_ 000197	Capparaceae	*Boscia angustifolia*	-0.89	-0.479	-0.701	-1	NA	0.671	0.292	0.005	-0.467	NA
seq_ 000220	Euphorbiaceae	*Euphorbia inaequilatera*	-1	0.889	0.258	-1	NA	-1	-1	0.889	0.727	0.871
seq_ 000265	Convolvulaceae	*Evolvulus alsinoides*	-1	-1	-1	-1	NA	-1	-1	-1	-1	-1
seq_ 000281	Malvaceae	*Sida ovata; Sida alba*	-0.279	0.387	0.061	0.061	NA	-1	-1	-0.279	-0.656	-0.354
seq_ 000289	Burseraceae	*Commiphora africana; Commiphora habessinica*	0.544	0.544	0.821	-1	NA	-1	-1	0.743	0.43	0.703
seq_ 000311	Fabaceae	*Vachellia nilotica; Vachellia seyal; Vachellia xanthophloea; Albizia sp.; Pterolobium stellatum*	0.864	0.386	0.386	-1	NA	0.917	0.79	0.839	0.616	NA
seq_ 000322	Amaranthaceae	*Psilotrichum elliotii*	-0.017	-0.611	-0.611	-1	NA	-1	-1	-0.611	-0.837	-0.661
seq_ 000450	Poaceae	*Aristida adscensionis*	-1	-0.588	-1	0.293	0.057	-1	NA	-1	NA	-1
seq_ 000473	Commelinaceae	*Commelina benghalensis*	0.151	-1	-1	-1	NA	-1	-1	-1	-1	-1
seq_ 000485	Acanthaceae	*Justicia*	-0.431	-0.114	-0.669	-0.669	NA	-0.669	-0.864	-0.431	-0.746	-0.497
seq_ 000515	Polygalaceae	*Polygala sphenoptera*	-1	-1	-1	-1	NA	-1	-1	-1	-1	-1
seq_ 000566	Acanthaceae	*Barleria spinisepala*	-0.591	-0.659	-0.903	-1	NA	-1	-1	-0.814	-0.929	-0.841
seq_ 000741	Lamiaceae	*Plectranthus sp.; Plectranthus barbatus; Plectranthus caninus; Plectranthus montanus; Plectranthus cylindraceus*	0.821	0.743	0.544	-1	NA	-1	-1	0.743	0.43	0.703
seq_ 000967	Verbenaceae	*Lantana viburnoides; Lippia javanica; Lantana camara*	-0.406	-0.406	-1	-1	NA	-1	-1	-0.083	-0.525	-0.165
seq_ 001294	Boraginaceae	*Heliotropium steudneri; Heliotropium zeylanicum*	-1	-1	-1	-1	NA	-1	-1	-1	-1	-1
seq_ 001501	Asteraceae	*Tripteris vaillantii*	-1	-0.193	-0.193	-1	NA	-1	-1	-1	-1	-1
seq_ 002188	Asteraceae	*Tagetes minuta*	-1	-1	-1	-1	NA	-1	-1	-1	-1	-1
seq_ 002993	Acanthaceae	*Barleria trispinosa; Barleria eranthemoides*	-1	-1	-1	-1	NA	-1	-1	0.021	-0.446	-0.063
seq_ 003220	Asparagaceae	*Asparagus falcatus*	-0.238	-1	-1	-1	NA	-0.53	-0.797	-0.04	-0.493	NA

seq_ 003570	Rubiaceae	*Coptosperma graveolens*	-1	-1	-1	-1	NA	0.497	0.046	-0.406	-0.73	-0.473
seq_ 003636	Poaceae	*Enneapogon cenchroides*	-1	0.061	-1	-1	-1	-1	NA	-1	NA	-1
seq_ 004441	Lamiaceae	*Ocimum filamentosum; Ocimum gratissimum*	-1	-1	-1	-1	NA	-1	-1	-1	-1	-1
seq_ 005900	Asparagaceae	*Chlorophytum gallabatense; Chlorophytum subpetiolatum; Asparagaceae sp.*	-0.279	0.387	-1	-1	NA	-1	-1	-0.279	-0.656	-0.354
seq_ 006918	Acanthaceae	*Blepharis maderaspatensis*	-1	-1	-1	-1	NA	-1	-1	-1	-1	-1
seq_ 007002	Oleaceae	*Jasminum fluminense*	0.258	0.545	0.258	-1	NA	0.878	0.701	-1	-1	-1
seq_ 007045	Apocynaceae	*Cynanchum viminale; Cynanchum gerrardii*	0.743	0.544	-1	-1	NA	0.821	0.58	0.821	0.58	0.792
seq_ 007106	Zygophyllaceae	*Balanites*	-0.193	-1	-1	-1	NA	-0.193	-0.602	-1	-1	-1
seq_ 016074	Acanthaceae	*Asystasia*	-1	-1	-1	-1	NA	-1	-1	-1	-1	-1
seq_ 017456	Fabaceae	*Medicago laciniata*	-1	-1	-1	-1	NA	-1	-1	-1	-1	-1
seq_ 021612	Convolvulaceae	*Ipomoea*	-1	-1	-1	-1	NA	-1	-1	-1	-1	-1
seq_ 044560	Zygophyllaceae	*Tribulus terrestris*	-0.348	-1	-1	-1	NA	-1	-1	-1	-1	-1
seq_ 069797	Malvaceae	*Hibiscus vitifolius*	-1	-1	-1	-1	NA	-1	-1	-1	-1	-1

Table S2. Model-selection results for selectivity by six dominant herbivore species at Mpala. Candidate models included additive combinations of 15 plant traits. All models with $\Delta AIC_c < 2$ are shown. For each response, conditionally averaged coefficients for models with $\Delta AIC_c < 2$ are presented in italics with standard errors in parentheses. Minerals are represented by elemental symbols; F_t is leaf toughness (force to tear).

	Inter-cept	Ca	Cu	Digest-ibility	Fe	Fiber	F_t	Height	K	Mg	Mo	Na	P	Protein	Spine-scent (yes)	Zn	R^2	df	AIC_c	delta	weight
Elephant (D_{ele})	-0.40		-0.18					0.30						0.13		-0.21	0.40	6	83.11	0.00	0.01
	-0.38		-0.20					0.32								-0.17	0.37	5	83.50	0.39	0.01
	-0.41		-0.18					0.28		-0.11				0.16		-0.16	0.41	7	84.32	1.21	0.01
	-0.40		-0.20					0.29		-0.18				0.16			0.38	6	84.43	1.32	0.01
	-0.37		-0.17				-0.06	0.31								-0.20	0.38	6	84.51	1.39	0.01
	-0.42		-0.19	0.09				0.33								-0.17	0.38	6	84.72	1.61	0.01
	-0.37		-0.18					0.35						0.13	-0.21	-0.24	0.40	7	84.88	1.77	0.00
	-0.37		-0.23					0.33									0.33	4	84.99	1.88	0.00
	-0.39 (0.07)		*-0.19 (0.09)*	*0.09 (0.09)*			*-0.06 (0.06)*	*0.31 (0.10)*		*-0.15 (0.10)*				*0.14 (0.08)*	*-0.21 (0.24)*	*-0.19 (0.10)*					
Impala (D_{imp})	-0.23		-0.17	0.19									-0.15				0.15	5	106.6	0.00	0.01
	-0.24			0.21									-0.15				0.11	4	107.4	0.78	0.00
	-0.22	0.12	-0.18	0.17									-0.15				0.18	6	107.5	0.85	0.00
	-0.17	0.14	-0.19										-0.13				0.14	5	107.5	0.91	0.00
	-0.18		-0.19										-0.12				0.11	4	107.6	0.92	0.00
	-0.19		-0.17										-0.13	0.12			0.13	5	108.22	1.54	0.00
	-0.19		-0.19	0.19									-0.16		-0.22		0.17	6	108.27	1.59	0.00
	-0.22		-0.18														0.06	3	108.30	1.62	0.00
	-0.21	0.13	-0.18														0.09	4	108.54	1.86	0.00
	-0.23	0.11		0.19									-0.15				0.13	5	108.54	1.86	0.00
	-0.11	0.16	-0.22										-0.15		-0.28		0.16	6	108.63	1.95	0.00
	-0.16	0.13	-0.21	0.17									-0.17		-0.28		0.19	7	108.66	1.98	0.00
	-0.20 (0.10)	*0.13 (0.10)*	*-0.19 (0.10)*	*0.19 (0.11)*									*-0.14 (0.08)*	*0.12 (0.10)*	*-0.26 (0.24)*						
Buffalo (D_{buf})	-0.21		-0.23										-0.11		-0.60		0.18	5	99.95	0.00	0.01
	-0.25		-0.22												-0.52		0.14	4	100.16	0.21	0.01
	-0.25		-0.24						-0.13						-0.58		0.17	5	100.91	0.96	0.00
	-0.26		-0.22							-0.12					-0.54		0.16	5	101.04	1.10	0.00
	-0.25		-0.20												-0.60	-0.13	0.16	5	101.07	1.12	0.00
	-0.21		-0.23							-0.10			-0.10		-0.61		0.19	6	101.29	1.34	0.00
	-0.23		-0.20		-0.17								-0.11		-0.60		0.19	6	101.47	1.53	0.00

	-0.25		-0.23			0.08									-0.47		0.16	5	101.52	1.57	0.00
	-0.21		-0.24			0.08							-0.10		-0.55		0.19	6	101.57	1.63	0.00
	-0.21		-0.25						-0.09				-0.09		-0.62		0.19	6	101.68	1.73	0.00
	-0.21		-0.22										-0.09		-0.64	-0.10	0.19	6	101.68	1.73	0.00
	-0.27		-0.18		-0.17										-0.52		0.15	5	101.70	1.75	0.00
	-0.19		-0.21				-0.05						-0.11		-0.60		0.19	6	101.81	1.86	0.00
	-0.26		-0.22								-0.09				-0.52		0.15	5	101.82	1.87	0.00
	-0.23		*-0.22*		*-0.17*	*0.08*	*-0.05*		*-0.11*	*-0.11*	*-0.09*		*-0.10*		*-0.57*	*-0.12*					
	(0.09)		*(0.10)*		*(0.18)*	*(0.09)*	*(0.06)*		*(0.11)*	*(0.10)*	*(0.11)*		*(0.07)*		*(0.23)*	*(0.12)*					
Zebra (D_{zeb})	-0.55				-0.32	0.32		-0.20	-0.16			0.38					0.62	7	48.47	0.00	0.02
	-0.62				-0.30	0.34		-0.21	-0.14								0.61	6	48.71	0.24	0.02
	-0.55				-0.32	0.29		-0.22	-0.13	-0.10		0.43					0.63	8	49.23	0.76	0.01
	-0.55				-0.32	0.32		-0.20		-0.15		0.38					0.61	7	49.70	1.24	0.01
	-0.53				-0.28	0.34	-0.05	-0.20	-0.18			0.43					0.63	8	49.83	1.37	0.01
	-0.62				-0.30	0.34		-0.21		-0.12							0.60	6	49.87	1.40	0.01
	-0.52				-0.27	0.31	-0.06	-0.22	-0.14	-0.11		0.50					0.64	9	50.14	1.67	0.01
	-0.63				-0.30	0.32		-0.22	-0.11	-0.08							0.61	7	50.18	1.72	0.01
	-0.57				*-0.31*	*0.32*	*-0.05*	*-0.21*	*-0.15*	*-0.11*		*0.42*									
	(0.07)				*(0.11)*	*(0.06)*	*(0.04)*	*(0.07)*	*(0.08)*	*(0.08)*		*(0.25)*									
Zebra (grass; $D_{zeb\text{-}grass}$)	-0.11						-0.13									-0.59	0.64	4	7.44	0.00	0.08
	-0.26						-0.14							-0.20		-0.56	0.73	5	7.57	0.12	0.07
	-0.45						-0.14			-0.49						-0.69	0.72	5	7.69	0.25	0.07
	0.12						-0.12										0.53	3	8.25	0.80	0.05
	-0.05						-0.13							-0.21			0.62	4	8.50	1.06	0.05
	-0.26						-0.15		-0.22							-0.71	0.70	5	8.85	1.41	0.04
	-0.77	-0.64					-0.13									-0.70	0.70	5	9.14	1.70	0.03
	0.10			-0.29			-0.11										0.60	4	9.20	1.75	0.03
	-0.21	*-0.64*		*-0.29*			*-0.13*		*-0.22*	*-0.49*				*-0.20*		*-0.63*					
	(0.32)	*(0.44)*		*(0.20)*			*(0.03)*		*(0.15)*	*(0.27)*				*(0.12)*		*(.30)*					
Giraffe (D_{gir})	-0.62		-0.15			-0.23		0.32	-0.36								0.43	6	92.60	0.00	0.02
	-0.57		-0.17			-0.26		0.41	-0.39						-0.36		0.45	7	92.96	0.36	0.02
	-0.62					-0.24		0.35	-0.34								0.41	5	93.28	0.68	0.02
	-0.59		-0.16			-0.23		0.29	-0.33				-0.07				0.44	7	93.96	1.37	0.01
	-0.54		-0.18			-0.25		0.38	-0.36				-0.07		-0.36		0.46	8	94.33	1.73	0.01
	-0.58					-0.26		0.42	-0.36						-0.27		0.41	6	94.53	1.93	0.01
	-0.59		*-0.16*			*-0.24*		*0.36*	*-0.36*				*-0.07*		*-0.36*						
	(0.08)		*(0.09)*			*(0.09)*		*(0.12)*	*(0.11)*				*(0.07)*		*(0.11)*						

Giraffe (nongrass; $D_{gir\text{-}non}$)	-0.58					0.21	-0.33				0.36	4	60.76	0.00	0.01
	-0.59		-0.13			0.19	-0.34				0.39	5	60.97	0.21	0.01
	-0.55	-0.13				0.20	-0.30				0.39	5	61.28	0.52	0.01
	-0.51		-0.15				-0.35	-0.11			0.38	5	61.56	0.80	0.01
	-0.55					0.17	-0.30	-0.08			0.38	5	61.70	0.94	0.01
	-0.56		-0.13			0.15	-0.31	-0.09			0.41	6	61.76	1.00	0.01
	-0.51	-0.14				0.16	-0.27	-0.09			0.41	6	61.87	1.11	0.01
	-0.61			-0.18		0.19	-0.33				0.38	5	62.11	1.34	0.00
	-0.46	-0.15					-0.30	-0.12			0.38	5	62.12	1.36	0.00
	-0.55		-0.15			0.25	-0.37			-0.25	0.41	6	62.33	1.56	0.00
	-0.50						-0.34	-0.12			0.34	4	62.34	1.58	0.00
	-0.45		-0.15		0.31		-0.31	-0.12			0.41	6	62.34	1.58	0.00
	-0.57	-0.10	-0.10			0.19	-0.32				0.40	6	62.53	1.77	0.00
	-0.55		-0.15				-0.40				0.33	4	62.54	1.78	0.00
	-0.55					0.26	-0.35			-0.19	0.37	5	62.63	1.87	0.00
	-0.48	-0.11	-0.12				-0.32	-0.12			0.40	6	62.72	1.95	0.00
	-0.54	*-0.13*	*-0.14*	*-0.18*	*0.31*	*0.20*	*-0.33*	*-0.10*		*-0.22*					
	(0.10)	*(0.10)*	*(0.09)*	*(0.17)*	*(0.24)*	*(0.11)*	*(0.11)*	*(0.07)*		*(0.23)*					
Dik-dik (D_{dik})	-0.39	0.23	-0.19			0.29	-0.23		0.19		0.42	7	97.89	0.00	0.02
	-0.35	0.24	-0.18			0.36					0.36	5	99.23	1.34	0.01
	-0.35	0.25	-0.21			0.37	-0.26		0.20	-0.29	0.43	8	99.25	1.37	0.01
	-0.36	0.30	-0.21			0.33	-0.16				0.38	6	99.31	1.42	0.01
	-0.37	0.18	-0.16			0.35			0.13		0.37	6	99.86	1.98	0.01
	-0.37	*0.24*	*-0.19*			*0.33*	*-0.22*		*0.18*	*-0.29*					
	(0.08)	*(0.10)*	*(0.10)*			*(0.11)*	*(0.12)*		*(0.11)*	*(0.27)*					
Dik-dik (nongrass; $D_{dik\text{-}non}$)	-0.45					0.23					0.10	3	73.12	0.00	0.00
	-0.42					0.18	-0.18				0.15	4	73.18	0.06	0.00
	-0.38						-0.23				0.10	3	73.46	0.33	0.00
	-0.45	0.18	-0.18				-0.28				0.19	5	73.78	0.65	0.00
	-0.40		-0.14				-0.24				0.13	4	74.01	0.88	0.00
	-0.46	0.14				0.19	-0.20				0.18	5	74.04	0.91	0.00
	-0.48	0.18	-0.16			0.16	-0.23				0.23	6	74.06	0.94	0.00
	-0.51				-0.35	0.22	-0.20				0.18	5	74.22	1.10	0.00
	-0.43		-0.12			0.16	-0.19				0.18	5	74.37	1.24	0.00
	-0.49					0.18	-0.20		0.14		0.17	5	74.47	1.35	0.00
	-0.41	0.13					-0.26				0.12	4	74.50	1.38	0.00

	-0.46		-0.10					0.22									0.12	4	74.54	1.42	0.00
	-0.42							0.19					-0.07				0.12	4	74.57	1.45	0.00
	-0.36								-0.19				-0.08				0.12	4	74.65	1.52	0.00
	-0.45							0.21								-0.10	0.12	4	74.65	1.53	0.00
	-0.48	0.10						0.24									0.12	4	74.67	1.55	0.00
	-0.52						-0.26	0.27									0.12	4	74.77	1.65	0.00
	-0.44								-0.25					0.13			0.12	4	74.81	1.69	0.00
	-0.36												-0.12				0.06	3	74.86	1.73	0.00
	-0.50							0.24						0.10			0.11	4	74.96	1.83	0.00
	-0.24								-0.27			0.56					0.11	4	75.06	1.94	0.00
	-0.43	0.20	-0.19					0.25	-0.27						-0.34		0.26	7	75.07	1.94	0.00
	-0.30							0.17	-0.21			0.50					0.16	5	75.08	1.95	0.00
	-0.43	*0.16*	*-0.15*				*-0.31*	*0.20*	*-0.22*			*0.53*	*-0.09*	*0.13*	*-0.34*	*-0.10*					
	(0.12)	*(0.12)*	*(0.11)*				*(0.31)*	*(0.12)*	*(0.12)*			*(0.64)*	*(0.08)*	*(0.13)*	*(0.27)*	*(0.11)*					
Dik-dik (≤1-m; $D_{dik-\leq 1-m}$)	-0.36	0.26	-0.21					0.43	-0.31					0.24	-0.50		0.48	8	90.60	0.00	0.02
	-0.43	0.23	-0.19					0.29	-0.26					0.23			0.45	7	91.25	0.65	0.02
	-0.38	*0.24*	*-0.20*					*0.37*	*-0.29*					*0.23*	*-0.50*						
	(0.09)	*(0.10)*	*(0.09)*					*(0.14)*	*(0.12)*					*(0.10)*	*(0.29)*						

Table S3. Model-selection results for plant responses to different herbivore-exclusion treatments (*e.g.,* TOTAL:OPEN is the effect size of excluding all herbivores larger than 5 kg relative to the case where all herbivores were allowed). Candidate models included additive combinations of species-specific herbivore selectivity estimates. All models included a random intercept for site. All models with ΔAICc < 2 are shown. For each response, conditionally averaged coefficients for models with $\Delta AIC_c < 2$ are presented in italics with standard errors in parentheses.

	Inter-cept	Elephant selectivity (D_{ele})	Impala selectivity (D_{imp})	Buffalo selectivity (D_{buf})	Zebra selectivity (D_{zeb})	Giraffe selectivity (D_{gir})	Dik-dik selectivity (D_{dik})*	R^2	df	AIC_c	delta	weight
TOTAL:OPEN	1.61	1.14						0.26	4	310.63	0.00	0.20
	1.30	1.05			-0.46			0.27	5	311.32	0.70	0.14
	1.47	1.27		-0.45				0.27	5	311.38	0.76	0.14
	1.47 (0.43)	*1.15 (0.30)*		*-0.45 (0.33)*	*-0.46 (0.33)*							
MEGA:OPEN	0.30	1.03			-0.64			0.28	5	282.60	0.00	0.25
	0.72	1.16						0.24	4	284.06	1.45	0.12
	0.44 (0.35)	*1.07 (0.26)*			*-0.64 (0.31)*							
MESO:OPEN	0.94	1.00			-0.60			0.33	5	292.40	0.00	0.25
	1.34	1.11						0.30	4	292.99	0.59	0.19
	1.11 (0.51)	*1.05 (0.29)*			*-0.60 (0.33)*							
MESO:MEGA	0.47							0.00	3	297.44	0.00	0.23
	0.32					-0.27		0.02	4	299.23	1.79	0.10
	0.43 (0.17)					*-0.27 (0.21)*						
TOTAL:MESO	0.26							0.02	3	195.94	0.00	0.44
	0.26 (0.12)											

*Results are quantitatively identical if ≤1-m selectivity is used in lieu of overall selectivity.

Table S4. Model selection results for plant responses to different herbivore-exclusion treatments (*e.g.,* TOTAL:OPEN is the effect size of excluding all herbivores larger than 5 kg relative to the case where all herbivores were allowed). Candidate models included additive combinations of elephant selectivity and 15 plant traits. All models included a random intercept for site. All models with $\Delta AIC_c < 2$ are shown. For each response, conditionally averaged coefficients for models with $\Delta AIC_c < 2$ are presented in italics with standard errors in parentheses.

	Intercept	Ca	Cu	Digestibility	D_{ele}	Fe	Fiber	F_t	Height	K	Mg	Mo	Na	P	Protein	Spinescent (yes)	Zn	R^2	df	AIC_c	delta	weight
TOTAL:OPEN	1.46				1.10	-0.77				0.52								0.34	6	268.96	0.00	0.01
	1.49				1.01					0.50	0.49						-0.68	0.35	7	269.00	0.03	0.01
	1.33				0.98	-0.86				0.71							-0.50	0.34	7	269.07	0.10	0.01
	1.60				1.06					0.51								0.33	5	269.07	0.10	0.01
	1.48				1.03	-0.92				0.67			0.84				-0.58	0.33	8	269.13	0.16	0.01
	1.51				1.07						0.66						-0.52	0.32	6	269.54	0.58	0.01
	1.49				0.95					0.69							-0.46	0.33	6	269.54	0.58	0.01
	1.61				1.16						0.45							0.32	5	269.67	0.71	0.00
	1.53				1.13	-0.79				0.49			0.34					0.33	7	269.76	0.80	0.00
	1.62				1.27					0.63	0.65				-0.40		-0.73	0.38	8	269.78	0.82	0.00
	1.62				0.98					0.66			0.71				-0.53	0.32	7	269.78	0.82	0.00
	1.55				1.03					0.49	0.46		0.37				-0.71	0.35	8	269.79	0.82	0.00
	1.65				1.08					0.49			0.27					0.32	6	269.85	0.88	0.00
	1.39				1.02	-0.53				0.54	0.41						-0.67	0.35	8	269.91	0.95	0.00
	1.45				0.94					0.61						0.51		0.33	6	269.93	0.97	0.00
	1.33				0.99	-0.74				0.61						0.48		0.33	7	270.03	1.07	0.00
	1.60				1.09						0.63		0.52				-0.56	0.31	7	270.06	1.10	0.00
	1.64				1.17						0.44		0.14					0.31	6	270.44	1.48	0.00
	1.47				1.05	-0.60				0.54	0.37		0.52				-0.71	0.35	9	270.58	1.62	0.00
	1.38				0.94	-0.89				0.74			0.93			0.43	-0.55	0.33	9	270.60	1.64	0.00
	1.52				0.97					0.58			0.41			0.55		0.32	7	270.63	1.67	0.00
	1.24				0.90	-0.83				0.77						0.37	-0.46	0.34	8	270.66	1.69	0.00
	1.60				1.14	-0.79							0.92					0.28	6	270.71	1.74	0.00
	1.51				1.21					0.79	0.61				-0.52	0.56	-0.66	0.38	9	270.71	1.74	0.00
	1.41				1.01	-0.76				0.58			0.48			0.52		0.33	8	270.73	1.77	0.00
	1.67				1.28					0.62	0.63		0.27		-0.40		-0.75	0.37	9	270.75	1.79	0.00
	1.54				1.17	-0.39					0.41							0.31	6	270.76	1.80	0.00
	1.53				1.05					0.56		-0.32						0.33	6	270.84	1.88	0.00
	1.73				1.10								0.85					0.27	5	270.85	1.89	0.00
	1.45				1.08	-0.30					0.63						-0.51	0.32	7	270.89	1.93	0.00

	1.38		0.86			0.76						0.41	-0.42	0.33	7	270.90	1.94	0.00
	1.51		*1.06*	*-0.74*		*0.60*	*0.53*	*-0.32*	*0.54*		*-0.43*	*0.48*	*-0.59*					
	(0.41)		*(0.30)*	*(0.63)*		*(0.26)*	*(0.26)*	*(0.28)*	*(0.91)*		*(0.26)*	*(0.48)*	*(0.31)*					
MEGA:OPEN	0.94		1.11	-1.08		0.40			1.50	-0.47				0.42	8	247.81	0.00	0.02
	0.79		0.99	-1.02		0.48			1.72	-0.42		0.66		0.44	9	247.88	0.07	0.02
	0.95		1.13	-1.12					1.91	-0.40				0.39	7	247.94	0.13	0.02
	0.96	-0.33	1.06	-0.90		0.59			2.11	-0.38		0.91		0.45	10	249.28	1.47	0.01
	0.86		1.05	-1.08					2.10	-0.36		0.42		0.39	8	249.42	1.61	0.01
	0.95		1.14	-0.84			0.29		1.51	-0.42				0.41	8	249.69	1.88	0.01
	0.90	*-0.33*	*1.08*	*-1.03*		*0.47*	*0.29*		*1.77*	*-0.42*		*0.66*						
	(0.28)	*(0.21)*	*(0.26)*	*(0.50)*		*(0.22)*	*(0.20)*		*(0.73)*	*(0.15)*		*(0.46)*						
MESO:OPEN	0.94		0.98	-0.93		1.03		-0.73			-0.50	1.15		0.50	9	246.73	0.00	0.02
	1.10		1.04	-0.96		0.98		-0.69	0.82		-0.54	1.22		0.50	10	247.01	0.28	0.02
	0.92		0.82	-0.89		0.70		-0.69				0.70		0.46	8	247.27	0.54	0.02
	1.13		1.04	-0.93		0.56		-0.65						0.46	7	247.29	0.56	0.02
	1.03		0.86	-0.90		0.65		-0.66	0.57			0.72		0.46	9	248.06	1.33	0.01
	1.23		1.08	-0.95		0.51		-0.63	0.50					0.45	8	248.10	1.37	0.01
	1.06		0.93			1.05		-0.74			-0.48	1.19		0.48	8	248.21	1.48	0.01
	1.04		0.78			0.74		-0.71				0.76		0.44	7	248.37	1.64	0.01
	1.28	-0.41	0.91	-0.76		0.79		-0.63	1.07			1.03		0.49	10	248.42	1.69	0.01
	1.40	-0.47	0.88			0.84		-0.64	1.09			1.12		0.47	9	248.50	1.77	0.01
	1.22		0.98			1.01		-0.71	0.75		-0.51	1.25		0.48	9	248.60	1.87	0.01
	1.09	*-0.44*	*0.95*	*-0.91*		*0.81*		*-0.68*	*0.79*		*-0.51*	*1.02*						
	(0.50)	*(0.22)*	*(0.30)*	*(0.50)*		*(0.31)*		*(0.22)*	*(0.82)*		*(0.25)*	*(0.54)*						
MESO:MEGA	0.33							-0.54						0.06	4	269.63	0.00	0.01
	0.03				0.62	0.49		-0.48	-1.25					0.15	7	270.08	0.45	0.01
	0.31							-0.52					-0.35	0.09	5	270.42	0.79	0.01
	0.15				0.70	0.47			-1.20					0.11	6	270.45	0.82	0.01
	0.08				0.57	0.57		-0.48	-0.92				-0.40	0.18	8	270.49	0.86	0.00
	0.26							-0.54	-0.35					0.06	5	270.50	0.87	0.00
	0.27				0.47	0.48		-0.47					-0.46	0.17	7	270.74	1.11	0.00
	0.20				0.64	0.55			-0.87				-0.40	0.14	7	270.89	1.26	0.00
	0.31				0.32			-0.46						0.08	5	270.90	1.27	0.00
	0.28							-0.62		0.30				0.09	5	270.93	1.30	0.00
	0.41				0.39									0.05	4	270.93	1.30	0.00
	0.46													0.00	3	271.02	1.39	0.00
	0.38				0.55	0.47							-0.45	0.13	6	271.04	1.41	0.00

	0.29	-0.22				-0.53					0.06	5	271.25	1.62	0.00
	0.20		0.34			-0.46	-0.54				0.09	6	271.52	1.89	0.00
	0.31					-0.52	0.00			-0.35	0.09	6	271.53	1.90	0.00
	0.31		0.42				-0.51				0.05	5	271.53	1.90	0.00
	0.44									-0.36	0.04	4	271.53	1.90	0.00
	0.40					-0.52			-0.43	-0.45	0.10	6	271.56	1.93	0.00
	0.30			0.31		-0.56				-0.48	0.12	6	271.59	1.96	0.00
	0.30		0.49	0.34		-0.47					0.12	6	271.62	1.99	0.00
	0.27	*-0.22*	*0.52*	*0.48*		*-0.51*	*-0.78*	*0.30*	*-0.43*	*-0.41*					
	(0.22)	*(0.51)*	*(.26)*	*(0.23)*		*(0.26)*	*(0.87)*	*(0.19)*	*(0.38)*	*(0.22)*					
TOTAL:MESO	0.22										0.00	3	227.89	0.00	0.02
	0.17						-0.27				0.00	4	229.09	1.20	0.01
	0.18				0.35					-0.52	0.08	5	229.49	1.60	0.01
	0.19									-0.27	0.03	4	229.58	1.69	0.01
	0.24	0.10									0.00	4	229.79	1.90	0.01
	0.20	*0.10*			*0.35*		*-0.27*			*-0.40*					
	(0.14)	*(0.47)*			*(0.17)*		*(0.62)*			*(0.25)*					

Table S5. Model-selection results for the frequency of occurrence (FOO) of plants in the diets of six dominant herbivore species at Mpala. Candidate models included additive combinations of 15 plant traits. All models with $\Delta AIC_c < 2$ are shown. For each model set, conditionally averaged coefficients for models with $\Delta AIC_c < 2$ are presented in italics with standard errors in parentheses.

	Inter-cept	Ca	Cu	Digest-ibility	Fe	Fiber	F_t	Height	K	Mg	Mo	Na	P	Protein	Spine-scent (yes)	Zn	R^2	df	AIC_c	delta	weight
Elephant FOO	0.27			0.10		0.14	-0.05					-0.04			0.27	-0.04	0.24	8	41.62	0.00	0.007
	0.27			0.09		0.17	-0.04					-0.04		0.05	0.28	-0.04	0.25	9	41.89	0.27	0.006
	0.26			0.08		0.14						-0.04		0.05	0.27	-0.04	0.23	8	42.31	0.68	0.005
	0.27			0.11		0.15	-0.04					-0.05			0.28		0.22	7	42.32	0.69	0.005
	0.26			0.10		0.18	-0.04					-0.05		0.05	0.30		0.23	8	42.51	0.89	0.005
	0.27			0.08		0.11						-0.04			0.26	-0.04	0.22	7	42.52	0.90	0.005
	0.27			0.10		0.15	-0.04		0.04			-0.04			0.28	-0.04	0.24	9	42.55	0.93	0.005
	0.26			0.09		0.15						-0.05		0.05	0.29		0.22	7	42.79	1.17	0.004
	0.27			0.09		0.13	-0.04								0.27	-0.05	0.21	7	42.97	1.35	0.004
	0.26			0.09		0.12						-0.05			0.28		0.20	6	43.07	1.44	0.003
	0.27			0.10		0.15	-0.04			0.03		-0.04			0.28	-0.05	0.24	9	43.14	1.52	0.003
	0.26			0.09		0.16	-0.04							0.05	0.28	-0.05	0.23	8	43.22	1.60	0.003
	0.27			0.11	-0.03	0.15	-0.04					-0.05			0.27		0.23	8	43.34	1.71	0.003
	0.27			0.08		0.12			0.04			-0.04			0.27	-0.04	0.23	8	43.35	1.73	0.003
	0.27			0.10		0.14	-0.05				0.02	-0.05			0.27	-0.05	0.24	9	43.36	1.74	0.003
	0.27			0.11		0.16	-0.04		0.03			-0.05			0.30		0.22	8	43.42	1.79	0.003
	0.26			0.08		0.13								0.05	0.28	-0.05	0.21	7	43.43	1.81	0.003
	0.26			0.09	-0.03	0.15						-0.05		0.06	0.28		0.22	8	43.43	1.81	0.003
	0.27			0.10		0.14	-0.04	0.02				-0.04			0.25	-0.04	0.24	9	43.44	1.82	0.003
	0.26			0.10	-0.03	0.18	-0.04					-0.05		0.05	0.28		0.24	9	43.44	1.82	0.003
	0.27	-0.03		0.11		0.14	-0.05					-0.05			0.29		0.22	8	43.47	1.85	0.003
	0.26	-0.03		0.10		0.17	-0.04					-0.05		0.05	0.30		0.24	9	43.54	1.91	0.003
	0.27			0.09		0.14	-0.04					-0.04	0.02		0.27	-0.04	0.24	9	43.56	1.93	0.003
	0.27	-0.02		0.10		0.13	-0.05					-0.05			0.27	-0.04	0.24	9	43.61	1.99	0.003
	0.27 (0.03)	*-0.03 (0.03)*		*0.09 (0.03)*	*-0.03 (0.03)*	*0.15 (0.04)*	*-0.04 (0.03)*	*0.02 (0.03)*	*0.04 (0.03)*	*0.03 (0.04)*	*0.02 (0.03)*	*-0.05 (0.02)*	*0.02 (0.03)*	*0.05 (0.04)*	*0.28 (0.08)*	*-0.04 (0.03)*					
Impal	0.31			0.13		0.15						-0.06			0.16		0.21	6	56.15	0.00	0.011
	0.32			0.14		0.18	-0.04					-0.06			0.17		0.22	7	56.59	0.44	0.009
	0.31			0.12		0.18						-0.06		0.04	0.17		0.22	7	57.35	1.20	0.006

	0.31	-0.03		0.12		0.14				-0.06		0.16		0.22	7	57.66	1.51	0.005
	0.32	-0.03		0.14		0.17	-0.04			-0.06		0.17		0.23	8	57.78	1.63	0.005
	0.32			0.12		0.15				-0.06		0.15	-0.02	0.22	7	57.80	1.65	0.005
	0.31			0.13		0.16			0.02	-0.06		0.17		0.22	7	57.84	1.69	0.005
	0.31			0.13	-0.02	0.15				-0.06		0.16		0.22	7	57.94	1.79	0.004
	0.31			0.13		0.16		0.02		-0.06		0.17		0.21	7	58.03	1.88	0.004
	0.31			0.14		0.20	-0.03			-0.06	0.03	0.18		0.23	8	58.05	1.90	0.004
	0.31		-0.02	0.13		0.16				-0.06		0.16		0.21	7	58.09	1.94	0.004
	0.31	*-0.03*	*-0.02*	*0.13*	*-0.02*	*0.16*	*-0.04*	*0.02*	*0.02*	*-0.06*	*0.04*	*0.17*	*-0.02*					
	(0.03)	*(0.03)*	*(0.03)*	*(0.03)*	*(0.03)*	*(0.04)*	*(0.03)*	*(0.04)*	*(0.03)*	*(0.03)*	*(0.04)*	*(0.08)*	*(0.03)*					
Buffalo FOO	0.32	-0.05		0.10		0.16	-0.05			-0.05				0.21	7	49.33	0.00	0.005
	0.32			0.11		0.18	-0.04			-0.04				0.20	6	49.84	0.52	0.004
	0.32			0.10		0.17	-0.05			-0.04			-0.04	0.21	7	50.00	0.67	0.004
	0.31	-0.05		0.11		0.17	-0.05			-0.05		0.09		0.22	8	50.11	0.79	0.004
	0.32	-0.06		0.11		0.17	-0.05		0.03	-0.05				0.22	8	50.28	0.96	0.003
	0.31			0.09		0.15				-0.04				0.18	5	50.40	1.07	0.003
	0.32	-0.05		0.10		0.18	-0.05			-0.05	0.04			0.22	8	50.45	1.13	0.003
	0.31	-0.04		0.09		0.13				-0.05				0.19	6	50.57	1.25	0.003
	0.32	-0.04		0.10		0.16	-0.05			-0.04			-0.03	0.22	8	50.57	1.25	0.003
	0.31			0.11		0.19	-0.05			-0.04		0.09		0.21	7	50.69	1.36	0.003
	0.32			0.08		0.14				-0.04			-0.04	0.19	6	50.70	1.37	0.003
	0.31	-0.06		0.11		0.18	-0.05		0.04	-0.05		0.11		0.23	9	50.70	1.38	0.003
	0.32			0.10		0.16	-0.04						-0.04	0.19	6	50.71	1.38	0.003
	0.33	-0.05		0.10		0.17	-0.05		0.04	-0.05			-0.04	0.23	9	50.79	1.47	0.002
	0.33			0.10		0.18	-0.05		0.04	-0.05			-0.05	0.22	8	50.92	1.60	0.002
	0.31	-0.05		0.10		0.20	-0.05			-0.05	0.04	0.10		0.23	9	50.94	1.61	0.002
	0.32	-0.04		0.10		0.16	-0.05							0.19	6	51.08	1.75	0.002
	0.32			0.11		0.17	-0.04							0.18	5	51.10	1.77	0.002
	0.32			0.10		0.20	-0.04			-0.04	0.04			0.20	7	51.10	1.78	0.002
	0.32			0.08		0.13							-0.04	0.17	5	51.21	1.88	0.002
	0.32			0.09		0.19	-0.04			-0.04	0.04		-0.04	0.22	8	51.28	1.96	0.002
	0.31			0.09		0.18				-0.04	0.04			0.19	6	51.29	1.96	0.002
	0.31	-0.04		0.09		0.16				-0.05	0.04			0.20	7	51.29	1.96	0.002
	0.32			0.11	-0.02	0.18	-0.04			-0.04				0.20	7	51.29	1.97	0.002
	0.31			0.10		0.18	-0.05			-0.04		0.08	-0.03	0.22	8	51.31	1.98	0.002

	0.32	*-0.05*	*0.1*	*-0.02*	*0.17*	*-0.05*				*0.04*	*-0.05*		*0.04*	*0.09*	*-0.04*					
	(0.03)	*(0.03)*	*(0.03)*	*(0.03)*	*(0.04)*	*(0.03)*				*(0.03)*	*(0.03)*		*(0.04)*	*(0.08)*	*(0.03)*					
Zebra FOO	0.21	-0.04	0.10		0.18						-0.05					0.37	6	-2.61	0.00	0.007
	0.20	-0.04	0.10		0.19						-0.05			0.09		0.38	7	-2.38	0.22	0.006
	0.20	-0.05	0.10		0.19					0.03	-0.05			0.10		0.39	8	-2.11	0.49	0.005
	0.21		0.10		0.18			-0.04			-0.05					0.37	6	-1.92	0.69	0.005
	0.21		0.10		0.20						-0.05					0.36	5	-1.91	0.69	0.005
	0.21	-0.04	0.10		0.17			-0.04		0.04	-0.05					0.38	8	-1.89	0.72	0.005
	0.21	-0.05	0.10		0.18					0.03	-0.05					0.37	7	-1.88	0.72	0.005
	0.21	-0.03	0.10		0.17			-0.03			-0.05					0.37	7	-1.86	0.75	0.004
	0.20		0.11		0.21						-0.04			0.09		0.36	6	-1.65	0.96	0.004
	0.22	-0.05	0.11		0.19					0.05	-0.06	-0.03				0.38	8	-1.54	1.07	0.004
	0.20	-0.05	0.11		0.19					0.05	-0.06	-0.03		0.09		0.39	9	-1.33	1.27	0.003
	0.20	-0.04	0.11		0.18			-0.03		0.04	-0.05			0.08		0.39	9	-1.33	1.28	0.003
	0.21		0.10		0.19						-0.04				-0.03	0.36	6	-1.16	1.45	0.003
	0.20	-0.04	0.10		0.18			-0.03			-0.05			0.07		0.38	8	-1.00	1.61	0.003
	0.21		0.10		0.18			-0.04		0.04	-0.05				-0.03	0.38	8	-0.98	1.63	0.003
	0.21		0.11		0.19			-0.05		0.03	-0.05					0.37	7	-0.97	1.63	0.003
	0.20		0.11		0.19			-0.03			-0.04			0.07		0.37	7	-0.92	1.68	0.003
	0.21	-0.03	0.10		0.18						-0.05				-0.02	0.37	7	-0.92	1.68	0.003
	0.21		0.10	-0.02	0.20						-0.05					0.36	6	-0.92	1.69	0.003
	0.21		0.10		0.18			-0.04			-0.04				-0.02	0.37	7	-0.90	1.70	0.003
	0.21	-0.04	0.11		0.18						-0.05	-0.02				0.37	7	-0.86	1.74	0.003
	0.21	-0.04	0.10		0.17			-0.04		0.04	-0.05				-0.02	0.39	9	-0.86	1.75	0.003
	0.21	-0.04	0.10		0.18					0.04	-0.05				-0.02	0.38	8	-0.78	1.82	0.003
	0.21	-0.03	0.10	-0.01	0.18						-0.05					0.37	7	-0.75	1.86	0.003
	0.21		0.10	-0.02	0.19			-0.04			-0.04					0.37	7	-0.72	1.88	0.003
	0.21	-0.05	0.11		0.18			-0.03		0.05	-0.06	-0.02				0.39	9	-0.63	1.97	0.002
	0.20	-0.05	0.11		0.19				0.02		-0.05			0.10		0.38	8	-0.62	1.99	0.002
	0.21	*-0.04*	*0.1*	*-0.02*	*0.18*			*-0.04*	*0.02*	*0.04*	*-0.05*	*-0.03*		*0.09*	*-0.02*					
	(0.02)	*(0.02)*	*(0.03)*	*(0.02)*	*(0.03)*			*(0.03)*	*(0.03)*	*(0.03)*	*(0.02)*	*(0.02)*		*(0.06)*	*(0.02)*					
Giraffe	0.19	-0.05	0.07				0.05	-0.06			-0.04			0.16		0.22	8	22.66	0.00	0.009
	0.19	-0.05	0.08				0.05	-0.06						0.16		0.21	7	23.30	0.64	0.006
	0.19	-0.06	0.07				0.04	-0.05			-0.04	-0.02		0.15		0.23	9	24.29	1.63	0.004
	0.20	-0.05	0.05					-0.06			-0.04			0.20		0.20	7	24.38	1.72	0.004

	0.19	-0.05		0.07				0.04	-0.06			-0.04			0.15	-0.02	0.22	9	24.54	1.88	0.003
	0.19	*-0.05*		*0.07*				*0.05*	*-0.06*			*-0.04*	*-0.02*		*0.16*	*-0.02*					
	(0.03)	*(0.03)*		*(0.03)*				*(0.02)*	*(0.03)*			*(0.02)*	*(0.03)*		*(0.07)*	*(0.02)*					
Dik-dik FOO	0.22			0.10		0.06		0.04				-0.03			0.19		0.17	7	26.54	0.00	0.004
	0.22			0.08		0.05						-0.04			0.22		0.15	6	26.56	0.01	0.004
	0.21			0.10		0.05		0.04							0.19		0.15	6	26.61	0.07	0.004
	0.22			0.09		0.08	-0.04					-0.04			0.23		0.17	7	26.62	0.08	0.004
	0.22			0.11		0.08	-0.03	0.03				-0.03			0.20		0.18	8	26.93	0.39	0.003
	0.22			0.11		0.07	-0.03	0.04							0.20		0.16	7	27.16	0.62	0.003
	0.22			0.08		0.05									0.23		0.13	5	27.23	0.69	0.003
	0.22			0.09		0.07	-0.03								0.24		0.15	6	27.46	0.91	0.003
	0.21			0.08				0.04							0.16		0.13	5	27.53	0.99	0.003
	0.22			0.07		0.05						-0.03			0.21	-0.03	0.16	7	27.63	1.09	0.002
	0.23			0.09		0.07	-0.04					-0.04			0.22	-0.03	0.17	8	27.65	1.11	0.002
	0.22			0.05											0.19	-0.04	0.13	5	27.65	1.11	0.002
	0.22			0.08		0.08						-0.04		0.03	0.23		0.16	7	27.78	1.24	0.002
	0.21			0.09		0.08		0.04				-0.03		0.03	0.19		0.17	8	27.90	1.36	0.002
	0.21			0.06											0.20		0.11	4	27.92	1.38	0.002
	0.22			0.07		0.04									0.21	-0.03	0.14	6	27.94	1.39	0.002
	0.22			0.09		0.04		0.04							0.18	-0.02	0.16	7	27.94	1.40	0.002
	0.21			0.10		0.07		0.04						0.03	0.20		0.16	7	27.94	1.40	0.002
	0.22			0.07				0.03							0.16	-0.03	0.14	6	27.96	1.42	0.002
	0.22			0.06								-0.03			0.19		0.13	5	27.98	1.44	0.002
	0.22			0.05								-0.03			0.18	-0.03	0.14	6	27.99	1.45	0.002
	0.22			0.08		0.07	-0.03								0.22	-0.03	0.16	7	28.09	1.55	0.002
	0.22			0.09		0.05		0.03				-0.03			0.18	-0.02	0.17	8	28.10	1.56	0.002
	0.21			0.07				0.04				-0.03			0.16		0.14	6	28.11	1.56	0.002
	0.22			0.08	-0.02	0.05						-0.04			0.21		0.16	7	28.11	1.57	0.002
	0.22			0.09		0.10	-0.03					-0.04		0.03	0.23		0.17	8	28.13	1.59	0.002
	0.22		-0.02	0.08		0.06						-0.04			0.21		0.16	7	28.18	1.64	0.002
	0.22			0.09	-0.02	0.08	-0.03					-0.04			0.22		0.17	8	28.37	1.83	0.002
	0.22			0.08		0.06				0.02		-0.04			0.23		0.15	7	28.39	1.85	0.002
	0.21			0.08		0.07								0.03	0.24		0.14	6	28.42	1.88	0.002
	0.22			0.10		0.07	-0.03	0.03							0.19	-0.02	0.17	8	28.42	1.88	0.002
	0.22			0.10		0.07	-0.03	0.03				-0.03			0.19	-0.02	0.18	9	28.44	1.90	0.002

	0.21		0.10		0.06		0.04		0.02					0.20		0.15	7	28.44	1.90	0.002
	0.22		0.10	-0.02	0.06		0.03				-0.03			0.18		0.17	8	28.44	1.90	0.002
	0.21		0.10	-0.02	0.05		0.04							0.19		0.15	7	28.46	1.91	0.002
	0.22		0.10		0.06		0.04		0.02		-0.03			0.19		0.17	8	28.48	1.94	0.002
	0.22	-0.02	0.09		0.08	-0.03					-0.04			0.22		0.17	8	28.53	1.99	0.002
	0.22	*-0.02*	*0.09*	*-0.02*	*0.06*	*-0.03*	*0.04*		*0.02*		*-0.03*		*0.03*	*0.2*	*-0.03*					
	(0.03)	*(0.03)*	*(0.03)*	*(0.03)*	*(0.04)*	*(0.02)*	*(0.03)*		*(0.03)*		*(0.02)*		*(0.03)*	*(0.08)*	*(0.02)*					

Table S6. Model-selection results for plant responses to different herbivore exclusion treatments (*e.g.,* TOTAL:OPEN is the effect size of excluding all herbivores larger than 5 kg relative to the case where all herbivores were allowed), using dietary frequency of occurrence (FOO) in lieu of selectivity as predictors. Candidate models included additive combinations of species-specific FOO. All models included a random intercept for site. All models with $\Delta AIC_c < 2$ are shown. For each response, conditionally averaged coefficients for models with $\Delta AIC_c < 2$ are presented in italics with standard errors in parentheses.

	Inter-cept	Elephant FOO	Impala FOO	Buffalo FOO	Zebra FOO	Giraffe FOO	Dik-dik FOO	R^2	df	AIC_c	delta	weight
TOTAL:OPEN	0.91	2.25			-2.15			0.21	5	348.02	0.00	0.849
	0.91 (0.48)	*2.25 (0.73)*			*-2.15 (0.63)*							
MEGA:OPEN	-0.20	2.42			-1.84			0.18	5	309.75	0.00	0.928
	-0.20 (0.35)	*2.42 (0.62)*			*-1.84 (0.53)*							
MESO:OPEN	0.71	2.21			-2.21			0.25	5	332.15	0.00	0.799
	0.71 (0.48)	*2.21 (0.67)*			*-2.21 (0.59)*							
MESO:MEGA	0.44							0.00	3	344.57	0.00	0.205
	0.31					0.35		0.01	4	345.97	1.40	0.102
	0.33				0.29			0.01	4	346.26	1.68	0.088
	0.48						-0.08	0.00	4	346.44	1.87	0.080
	0.50		-0.56			0.73		0.02	5	346.51	1.94	0.078
	0.39	0.10						0.00	4	346.52	1.95	0.077
	0.41 (0.23)	*0.10 (0.44)*	*-0.56 (0.59)*		*0.29 (0.38)*	*0.51 (0.54)*	*-0.08 (0.47)*					
TOTAL:MESO	0.23							0.02	3	228.82	0.00	0.385
	0.23 (0.11)											

Table S7. Model selection results for relative read abundance (RRA) of plants in the diets of six dominant herbivore species at Mpala. Candidate models included additive combinations of 15 plant traits. All models with $\Delta AIC_c < 2$ are shown. For each response, conditionally averaged coefficients for models with $\Delta AIC_c < 2$ are presented in italics with standard errors in parentheses.

	Inter-cept	Ca	Cu	Digest-ibility	Fe	Fiber	F_t	Height	K	Mg	Mo	Na	P	Protein	Spine-scent (yes)	Zn	R^2	df	AIC_c	delta	weight
Elephant RRA	0.00								-0.01						0.03		0.13	4	-406.65	0.00	0.00
	0.00														0.03		0.11	3	-406.44	0.21	0.00
	0.00								-0.01					0.01	0.03		0.15	5	-406.39	0.27	0.00
	0.00						0.00		-0.01					0.01	0.03		0.16	6	-406.18	0.47	0.00
	0.00						0.00								0.03		0.13	4	-405.67	0.99	0.00
	0.00														0.03	0.00	0.12	4	-405.60	1.05	0.00
	0.00								-0.01						0.03	0.00	0.14	5	-405.60	1.05	0.00
	0.00							0.00							0.03		0.12	4	-405.50	1.15	0.00
	0.00			0.00					-0.01						0.03		0.14	5	-405.38	1.28	0.00
	0.00							0.00	-0.01						0.03		0.14	5	-405.34	1.32	0.00
	0.00						0.00	0.00	-0.01					0.01	0.03		0.17	7	-405.29	1.37	0.00
	0.00								-0.01					0.01	0.03	0.00	0.16	6	-405.25	1.40	0.00
	0.00					0.00			-0.01					0.01	0.03		0.16	6	-405.22	1.43	0.00
	0.00						0.00		0.00						0.03		0.14	5	-405.22	1.43	0.00
	0.00	0.00													0.03		0.12	4	-405.20	1.45	0.00
	0.00			0.01				0.00	-0.01						0.03		0.16	6	-405.20	1.45	0.00
	0.00							0.00	-0.01					0.01	0.03		0.15	6	-405.11	1.54	0.00
	0.00										0.00				0.03		0.12	4	-405.00	1.66	0.00
	0.00						0.00	0.00							0.03		0.14	5	-404.92	1.73	0.00
	0.00			0.00				0.00							0.03		0.14	5	-404.91	1.74	0.00
	0.00									0.00					0.03		0.12	4	-404.82	1.83	0.00
	0.00			0.00											0.03		0.12	4	-404.80	1.86	0.00
	0.00	0.00							0.00						0.03		0.13	5	-404.73	1.92	0.00
	0.00	0.00							-0.01					0.01	0.03		0.15	6	-404.73	1.92	0.00
	0.00								-0.01		0.00				0.03		0.13	5	-404.71	1.95	0.00
	0.00					0.00									0.03		0.12	4	-404.68	1.98	0.00
	0.00 (0.00)	*0.00 (0.00)*		*0.00 (0.00)*		*0.00 (0.00)*	*0.00 (0.00)*	*0.00 (0.00)*	*-0.01 (0.00)*	*0.00 (0.00)*	*0.00 (0.00)*			*0.01 (0.00)*	*0.03 (0.01)*	*0.00 (0.00)*					
I	0.00			0.01				0.00	-0.01						0.01		0.16	6	-476.82	0.00	0.01

	0.00			0.00					-0.01				0.02		0.14	5	-476.07	0.75	0.00
	0.00			0.01		0.01		0.00	-0.01			0.01	0.02		0.19	8	-475.46	1.36	0.00
	0.01			0.01				0.00	-0.01				0.01	0.00	0.17	7	-475.45	1.36	0.00
	0.00			0.01				0.00	-0.01			0.00	0.01		0.17	7	-475.40	1.42	0.00
	0.00			0.01		0.00		0.00	0.00				0.02		0.17	7	-475.23	1.59	0.00
	0.01			0.00					-0.01				0.02	0.00	0.15	6	-475.19	1.63	0.00
	0.00			0.01		0.01			-0.01			0.01	0.02		0.17	7	-475.15	1.66	0.00
	0.00			0.00					-0.01			0.00	0.02		0.15	6	-474.99	1.82	0.00
	0.00	0.00		0.01				0.00	0.00				0.01		0.17	7	-474.83	1.99	0.00
	0.00 (0.00)	*0.00* (0.00)		*0.01* (0.00)		*0.00* (0.00)		*0.00* (0.00)	*-0.01* (0.00)			*0.00* (0.00)	*0.02* (0.01)	*0.00* (0.00)					
Buffalo RRA	0.01					0.01		0.00							0.17	4	-553.32	0.00	0.00
	0.01				0.00	0.01		0.00							0.19	5	-553.16	0.17	0.00
	0.01					0.01		0.00						0.00	0.19	5	-552.92	0.41	0.00
	0.01			0.00		0.01									0.17	4	-552.68	0.64	0.00
	0.01					0.01		0.00			0.00				0.18	5	-552.41	0.91	0.00
	0.01					0.01					0.00				0.17	4	-552.30	1.02	0.00
	0.01			0.00		0.01		0.00							0.18	5	-552.24	1.08	0.00
	0.01					0.01									0.15	3	-552.17	1.15	0.00
	0.01					0.01		0.00		0.00					0.18	5	-552.07	1.25	0.00
	0.01					0.01	0.00	0.00							0.18	5	-552.06	1.26	0.00
	0.01					0.01	0.00	0.00						0.00	0.20	6	-552.00	1.32	0.00
	0.01			0.00		0.01	0.00								0.18	5	-551.91	1.41	0.00
	0.01		0.00			0.01		0.00							0.18	5	-551.85	1.47	0.00
	0.01				0.00	0.01		0.00			0.00				0.19	6	-551.82	1.51	0.00
	0.01				0.00	0.01	0.00	0.00							0.19	6	-551.80	1.52	0.00
	0.01					0.01		0.00			0.00			0.00	0.19	6	-551.79	1.53	0.00
	0.01			0.00	0.00	0.01		0.00							0.19	6	-551.77	1.55	0.00
	0.01				0.00	0.01		0.00		0.00					0.19	6	-551.76	1.56	0.00
	0.01			0.00		0.01					0.00				0.18	5	-551.66	1.66	0.00
	0.01			0.00		0.01	0.00	0.00							0.19	6	-551.66	1.66	0.00
	0.01					0.01		0.00					0.00		0.18	5	-551.60	1.72	0.00
	0.01			0.00	0.00	0.01									0.18	5	-551.58	1.74	0.00
	0.01	0.00				0.01		0.00							0.18	5	-551.41	1.92	0.00
	0.01				0.00	0.01		0.00						0.00	0.19	6	-551.38	1.94	0.00

	0.01					0.01		0.00		0.00				0.00	0.19	6	-551.33	1.99	0.00
	0.01					0.01								0.00	0.16	4	-551.33	2.00	0.00
	0.01	*0.00*	*0.00*	*0.00*	*0.00*	*0.01*	*0.00*	*0.00*		*0.00*	*0.00*		*0.00*	*0.00*					
	(0.00)	*(0.00)*	*(0.00)*	*(0.00)*	*(0.00)*	*(0.00)*	*(0.00)*	*(0.00)*		*(0.00)*	*(0.00)*		*(0.01)*	*(0.00)*					
Zebra RRA	0.01					0.01		0.00		0.00					0.24	5	-478.39	0.00	0.00
	0.01					0.01		0.00						0.00	0.24	5	-478.15	0.24	0.00
	0.01					0.01		0.00							0.23	4	-478.12	0.27	0.00
	0.01					0.01		0.00		0.00				0.00	0.25	6	-477.88	0.51	0.00
	0.01	0.00				0.01		0.00		0.00					0.25	6	-477.84	0.55	0.00
	0.01					0.01		0.00				0.00		0.00	0.25	6	-477.77	0.63	0.00
	0.01				0.00	0.01		0.00		0.00					0.25	6	-477.56	0.83	0.00
	0.01					0.01		0.00				0.00			0.24	5	-477.47	0.92	0.00
	0.01				0.00	0.01		0.00							0.24	5	-477.46	0.93	0.00
	0.01	0.00				0.01		0.00							0.24	5	-477.44	0.95	0.00
	0.01					0.01		0.00		0.00		0.00			0.25	6	-477.40	1.00	0.00
	0.01					0.01		0.00		0.00		0.00		0.00	0.26	7	-477.12	1.27	0.00
	0.01			0.00		0.01		0.00							0.23	5	-476.97	1.42	0.00
	0.01			0.00		0.01		0.00		0.00					0.25	6	-476.90	1.49	0.00
	0.01	0.00				0.01		0.00		0.00		0.00			0.26	7	-476.81	1.59	0.00
	0.01	0.00				0.01		0.00				0.00			0.25	6	-476.76	1.63	0.00
	0.01				0.00	0.01		0.00				0.00			0.25	6	-476.76	1.63	0.00
	0.01			0.00		0.01									0.22	4	-476.59	1.80	0.00
	0.01					0.01		-0.01		0.00			0.00		0.25	6	-476.58	1.81	0.00
	0.01	0.00				0.01		0.00						0.00	0.25	6	-476.57	1.82	0.00
	0.01				0.00	0.01		0.00		0.00		0.00			0.26	7	-476.52	1.88	0.00
	0.01	0.00				0.01		0.00		0.00				0.00	0.26	7	-476.46	1.93	0.00
	0.01			0.00		0.01		0.00				0.00			0.24	6	-476.43	1.96	0.00
	0.01					0.01		0.00					0.00		0.23	5	-476.42	1.98	0.00
	0.01					0.01	0.00	0.00		0.00					0.24	6	-476.41	1.98	0.00
	0.01	*0.00*		*0.00*	*0.00*	*0.01*	*0.00*	*0.00*		*0.00*		*0.00*	*0.00*	*0.00*					
	(0.00)	*(0.00)*		*(0.00)*	*(0.00)*	*(0.00)*	*(0.00)*	*(0.00)*		*(0.00)*		*(0.00)*	*(0.01)*	*(0.00)*					
Giraffe	0.00			0.01				0.01	-0.01				0.03		0.23	6	-400.04	0.00	0.01
	0.00			0.01				0.01	-0.01			0.01	0.03		0.24	7	-399.90	0.14	0.01
	0.00			0.01			0.00	0.01	-0.01			0.01	0.03		0.25	8	-398.79	1.25	0.01
	0.00	0.00		0.01				0.01	-0.01			0.01	0.03		0.24	8	-398.49	1.55	0.00

	0.00	0.00	0.01				0.01	-0.01			0.03	0.23	7	-398.24	1.80	0.00
	0.00						0.01	-0.01		0.01	0.03	0.21	6	-398.16	1.88	0.00
	0.00		0.01				0.01	-0.01	0.00		0.03	0.23	7	-398.06	1.97	0.00
	0.00	*0.00*	*0.01*			*0.00*	*0.01*	*-0.01*	*0.00*	*0.01*	*0.03*					
	(0.00)	*(0.00)*	*(0.00)*			*(0.00)*	*(0.00)*	*(0.00)*	*(0.00)*	*(0.00)*	*(0.01)*					
Dik-dik RRA	0.00		0.01				0.01	-0.01		0.01	0.02	0.26	7	-454.22	0.00	0.01
	0.00		0.01				0.01	-0.01			0.02	0.23	6	-453.03	1.19	0.01
	0.00		0.01		0.00		0.01	-0.01		0.01	0.02	0.26	8	-452.92	1.30	0.01
	0.00		0.01	0.00			0.01	-0.01		0.01	0.02	0.26	8	-452.54	1.68	0.01
	0.00		0.01			0.00	0.01	-0.01		0.01	0.02	0.26	8	-452.35	1.87	0.01
	0.00	*0.00*	*0.01*	*0.00*	*0.00*	*0.00*	*0.01*	*-0.01*		*0.01*	*0.02*					
	(0.00)	*(0.00)*	*(0.00)*	*(0.00)*	*(0.00)*	*(0.00)*	*(0.00)*	*(0.00)*		*(0.00)*	*(0.01)*					

Table S8. Model selection results for plant responses to different herbivore exclusion treatments (*e.g.,* TOTAL:OPEN is the effect size of excluding all herbivores larger than 5 kg relative to the case where all herbivores were allowed), using dietary relative read abundance (RRA) in lieu of selectivity as predictors. Candidate models included additive combinations of species-specific RRA. All models included a random intercept for site. All models with $\Delta AIC_c < 2$ are shown. For each model set, conditionally averaged coefficients for models with $\Delta AIC_c < 2$ are presented in italics with standard errors in parentheses.

	Inter-cept	Elephant RRA	Impala RRA	Buffalo RRA	Zebra RRA	Giraffe RRA	Dik-dik RRA	R^2	df	AIC_c	delta	weight
TOTAL:OPEN	1.40		5.91	-11.23				0.15	5	343.76	0.00	0.24
	1.40		4.39		-8.11			0.15	5	344.35	0.59	0.18
	1.44	3.00		-9.70				0.15	5	345.36	1.61	0.11
	1.43	2.48			-7.93			0.15	5	345.47	1.72	0.10
	1.41 (0.43)	*2.75 (2.85)*	*5.26 (4.33)*	*-10.76 (7.11)*	*-8.04 (5.05)*							
MEGA:OPEN	0.52	4.89			-10.62			0.12	5	309.66	0.00	0.25
	0.53		5.89		-10.90			0.11	5	310.27	0.61	0.18
	0.52	5.40		-10.72				0.10	5	310.89	1.23	0.13
	0.51		7.30	-12.02				0.09	5	311.08	1.41	0.12
	0.52 (0.26)	*5.07 (2.50)*	*6.45 (3.84)*	*-10.74 (4.92)*	*-10.74 (4.92)*							
MESO:OPEN	1.14		5.56	-10.84				0.18	5	330.94	0.00	0.19
	1.14	4.37		-9.98				0.19	5	330.96	0.01	0.19
	1.10	3.88			-7.10			0.18	5	331.84	0.90	0.12
	1.11		4.17		-7.23			0.17	5	332.10	1.15	0.11
	1.15			-8.27		3.19		0.18	5	332.18	1.23	0.10
	1.12				-6.04	2.79		0.18	5	332.89	1.95	0.07
	1.13 (0.44)	*4.18 (2.68)*	*5.06 (4.11)*	*-9.96 (6.68)*	*-6.89 (5.14)*	*3.03 (2.59)*						
MESO:MEGA	0.30		-0.09		9.21			0.05	5	335.35	0.00	0.22
	0.32				9.03		-0.76	0.05	5	335.59	0.25	0.19
	0.30	0.01			9.20			0.05	5	336.12	0.78	0.15
	0.29				9.32	0.50		0.05	5	336.12	0.78	0.15
	0.30				9.20			0.05	4	337.33	1.98	0.08
	0.31 (0.16)	*0.01 (2.24)*	*-0.09 (3.31)*		*9.18 (4.31)*	*0.50 (2.18)*	*-0.76 (2.82)*					
TOTAL:MESO	0.26	-1.34		0.35				0.03	5	225.60	0.00	0.10

	0.26			-0.15			-1.20	0.03	5	225.64	0.04	0.10
	0.27			-0.16		-1.30		0.03	5	225.67	0.07	0.10
	0.24		-0.39	-0.05				0.02	5	225.68	0.08	0.10
	0.29				-1.37		-1.34	0.03	5	225.89	0.29	0.09
	0.29				-1.41	-1.39		0.04	5	225.91	0.30	0.09
	0.29	-1.29			-0.98			0.04	5	226.06	0.46	0.08
	0.26		-0.33		-1.03			0.02	5	226.16	0.56	0.08
	0.24			-0.24				0.02	4	226.78	1.18	0.06
	0.25				-1.07			0.02	4	227.17	1.57	0.05
	0.27<br>*(0.13)*	*-1.32*<br>*(1.37)*	*-0.36*<br>*(2.08)*	*-0.03*<br>*(3.40)*	*-1.19*<br>*(2.54)*	*-1.34*<br>*(1.32)*	*-1.27*<br>*(1.71)*					
