# Supplementary material for: Impacts of large herbivores on savanna plant communities: Predictive models of herbivore selectivity and plant response
Source: Ecology. 2026 Jul 23;107(7):e70445. doi: 10.1002/ecy.70445 (PMC13394317; doi:10.1002/ecy.70445)
Supplement: Supplementary file 2 — Appendix S2: [file ECY-107-e70445-s001.pdf]

APPENDIX S2

Impacts of large herbivores on savanna plant communities: Predictive models of herbivore selectivity and plant response

Joel O. Abraham, Maria Stahl, Samson Kurukura, Abdikadir Ali Hassan, Jacob R. Goheen, Todd M. Palmer, Tyler R. Kartzinel, Robert M. Pringle

Ecology

This Appendix contains:

Supplementary methods

SUPPLEMENTARY METHODS

Cross-validation of vegetation survey data

Part 1: Validating the canopy intercept (pin hit) methodology

Because the canopy intercept method has traditionally been used to estimate specifically *herbaceous* biomass (Frank and McNaughton 1990), we sought to evaluate its ability to represent availability for diverse plant functional types. To assess whether our sampling methodology produced representative data on plant abundance across plant functional types, we first reanalyzed classic data from Frank and McNaughton (1990). We used PlotDigitizer software to estimate the x – y values of data in their Fig. 1 and ran regressions of overall biomass as a function of pin hits (ignoring growth-form distinctions). We then converted these data to rank abundances and did the same. Ignoring plant growth forms resulted in a poorer fit than the group-specific regressions shown in Frank and McNaughton (1990), but not overwhelmingly (R^2 = 0.67 vs. 0.80 – 0.95). Conversion to ranks resulted in a 1:1 correlation with tight fit (R^2 = 0.83, Spearman's ρ = 0.91), indicating that relative biomass was almost perfectly predicted by ranked pin hits.

Next, we compared our 'giant pin' canopy intercept data to vegetation data collected from the UHURU plots using more conventional methods, including woody plant censuses and understory vegetation surveys, available from Alston et al. (2022).

Woody plant censuses were conducted in the UHURU plots from 2009 to 2019. During these surveys, all woody plants within the central 60 × 60-m (total survey area = 3600-m^2 plot^{-1}) monitoring grids of UHURU plots were identified, counted, and assigned to height classes. We used data from the two surveys conducted in 2017–2018, as these years most closely aligned with our giant pin surveys. Note that woody plant census data were missing for the S3MESO plot in 2017, which may have introduced some error. To facilitate comparison, we subsetted our giant pin-hit dataset to include only species also recorded in the woody plant censuses. We averaged the number of individuals of each species per plot and then compared the mean number of individuals of each species per plot (from the census) to the total number of pin hits per species across plots (summed across the three canopy intercept surveys) using linear modeling (**Fig. S1a**). We then converted the data to rank abundances and compared species ranks across the two datasets using linear modeling and calculated Spearman's rank order correlation (**Fig. S1d**).

For additional validation, two different understory vegetation datasets were available from Alston et al. (2022): (1) quadrat surveys and (2) pin-frame surveys. For quadrat surveys, 1-m^2 quadrats were placed at each of the 49 rebar stakes within the central 60 × 60 m monitoring grids of the UHURU plots. Percent cover was estimated for each plant species within each quadrat. These surveys included seedlings and saplings of overstory species as part of the understory. To improve comparability, we subsetted our canopy intercept data to only pin hits ≤1-m in height, to better represent understory vegetation. Twenty semiannual surveys were conducted between October

2008 and March 2019; as above, we used data from the four 2017–2018 surveys to match the timing of our canopy intercept surveys. For each species, we summed the number of pin hits and averaged percent cover across plots and surveys, and then compared these two abundance metrics using linear modeling (**Fig. S1b**). We also converted the data to rank abundances and compared species ranks across the two datasets using linear modeling and calculated Spearman's rank order correlation (**Fig. S1e**).

Pin-frame understory surveys were also conducted semiannually between October 2008 and March 2019; we again used data from the four 2017–2018 surveys to match the timing of our canopy intercept surveys. Within smaller 0.5 × 0.5-m (0.25-m^2) quadrats, the number of intercepts between vegetation and a 10-pin frame, likewise placed at each of the 49 rebar stakes within each UHURU plot, were recorded. We again subsetted our canopy intercept data to only pin hits ≤1-m in height and, for each species, summed the number of giant pin hits per plot and averaged the number of pin-frame hits across plots and surveys, and then compared the two abundance metrics using linear modeling (**Fig. S1c**). We again converted the data to rank abundances and compared species ranks across the two datasets using linear modeling and calculated Spearman's rank order correlation (**Fig. S1f**).

For all three dataset comparisons, our giant pin canopy intercept method yielded comparable data on plant abundance to other methods of surveying plant abundance. (1) We found that woody plant abundance (mean number of individuals per plot) was significantly positively correlated with total pin hits and explained the majority of variation in total pin hits (linear model; $F_{1,29} = 44.98$, $R^2 = 0.59$, $P < 0.001$; **Fig. S1**). (2) Similarly, mean percent cover from understory quadrat surveys was significantly positively correlated with ≤1-m pin hits and explained 95% of the variation (linear model; $F_{1,97} = 1806$, $R^2 = 0.95$, $P < 0.001$; **Fig. S1**); (3) the same was true with mean understory pin-frame hits (linear model; $F_{1,88} = 2643$, $R^2 = 0.97$, $P < 0.001$; **Fig. S1**). Rank abundances of plant species were correlated across all three comparisons: Spearman's rank order correlation indicated that species ranks within the giant pin-hit dataset were significantly correlated to those in the woody plant survey ($\rho = 0.69$, $S = 1509.3$, $P < 0.001$), the understory quadrat survey ($\rho = 0.73$, $S = 43749$, $P < 0.001$), and the understory pin frame survey ($\rho = 0.73$, $S = 32534$, $P < 0.001$). These results suggest that our canopy intercept methodology effectively captured variation in plant abundance across plant functional types.

Part 2: Validating landscape-wide availability survey

Because our Mpala-wide vegetation availability survey was less intensive than the plot-based canopy intercept surveys, we sought to verify that the broader-scale survey accurately reflected patterns of plant abundance. To do so, we compared landscape-wide availability estimates to data from the OPEN UHURU plots, where canopy intercept sampling was more intensive. While perfect congruence was not expected given the clustered spatial distribution of the OPEN plots versus the broader range of habitats represented in the park-wide survey (**Fig. 1**), we anticipated

that general patterns of species abundance would be consistent, as patterns of plant abundance tend to be broadly similar across the landscape.

We calculated the relative availability of each plant species in the OPEN plots by dividing the total number of pin hits per species by the total number of pin hits across all species. We then compared these values to relative abundance estimates from the park-wide survey using linear modeling (**Fig. S2a**). We repeated this comparison with taxonomic resolution coarsened to molecular operational taxonomic units (mOTUs), as this was the resolution of downstream analyses presented in the main text (**Fig. S2b**). For both taxonomic resolutions, we additionally converted the data to rank abundances and compared ranks across the two datasets using linear modeling and calculated Spearman's rank order correlation (**Fig. S2c-d**).

We found a significant positive correlation between relative abundances estimated from the park-wide survey and those from the OPEN plots. This correlation was significant at both the species-level (linear model; $F_{1,61} = 50.12$, $R^2 = 0.44$, $P < 0.001$; **Fig. S2**) and at the mOTU-level (linear model; $F_{1,56} = 48.29$, $R^2 = 0.45$, $P < 0.001$; **Fig. S2**). Furthermore, Spearman's rank order correlation indicated that species ranks were significantly correlated across the two datasets, both at the species-level ($\rho = 0.65$, $S = 14754$, $P < 0.001$) and at the mOTU-level ($\rho = 0.65$, $S = 11498$, $P < 0.001$). These patterns suggest that the park-wide availability survey reliably captured broad-scale patterns of plant abundance.

Literature cited

Alston, J. M., C. G. Reed, L. M. Khasoha, B. R. P. Brown, G. Busienei, N. Carlson, T. C. Coverdale, M. Dudenhoeffer, M. A. Dyck, J. Ekeno, A. A. Hassan, R. Hohbein, R. P. Jakopak, B. Kimiti, S. Kurukura, P. Lokeny, A. M. Louthan, S. Musila, P. M. Musili, T. Tindall, S. Weiner, T. R. Kartzinel, T. M. Palmer, R. M. Pringle, and J. R. Goheen. 2022. Ecological consequences of large herbivore exclusion in an African savanna: 12 years of data from the UHURU experiment. *Ecology* 103:e3649.

Frank, D. A., and S. J. McNaughton. 1990. Aboveground biomass estimation with the canopy intercept method: A plant growth form caveat. *Oikos* 57:57–60.
